# Study Design Complexity and Participant Completion in Dietary Trials for Inflammatory Bowel Disease: A Systematic Review and Metaresearch Study

**DOI:** 10.1016/j.advnut.2026.100614

**Published:** 2026-03-11

**Authors:** Laura Gregersen, Caroline Moos, Zainab Hikmat, Nathalie Fogh Rasmussen, Sofie Ronja Petersen, Berit Lilienthal Heitmann, þórhallur Ingi Halldórsson, Vibeke Andersen, Robin Christensen

**Affiliations:** 1 Molecular Diagnostics and Clinical Research Unit, Department of Regional Health Research, University of Southern Denmark, Odense, Denmark; 2 The Faculty of Health Sciences, Department of Regional Health Research, University of Southern Denmark, Odense, Denmark; 3 Section for Biostatistics and Evidence-Based Research, the Parker Institute, Bispebjerg and Frederiksberg Hospital, Copenhagen, Denmark; 4 Department of Clinical Research, University Hospital of Southern Denmark, Hospital Sønderjylland, Aabenraa Denmark; 5 Research Unit for Diet and Health, the Parker Institute, Bispebjerg and Frederiksberg Hospital, Copenhagen, Denmark; 6 Section for General Practice, Department of Public Health, University of Copenhagen, Copenhagen, Denmark; 7 The Boden Group, Charles Perkins Centre, University of Sydney, Sydney, New South Wales, Australia; 8 Faculty of Food and Science, School of Health Sciences, University of Iceland, Reykjavik, Iceland; 9 Research Unit of Rheumatology, Department of Clinical Research, University of Southern Denmark, Odense University Hospital, Denmark

**Keywords:** inflammatory bowel disease, IBD, dietary intervention trial design, trial completion rate, metaresearch, dietary adherence

## Abstract

Dietary intervention trials in inflammatory bowel disease (IBD) are difficult to implement due to poor adherence, despite their increasing importance for disease management. We conducted a systematic review to evaluate completion rates of dietary intervention trials recruiting patients with IBD and assess completion rate associations with trial design features. We systematically searched MEDLINE (Ovid), Embase (Ovid) and CINAHL (EBSCO) databases on 13 May, 2024 for randomized controlled trials examining dietary or macronutrient supplementary intake effects in patients with IBD, excluding enteral and parenteral nutrition trials. The flow of participants and prespecified trial design features were extracted. Completion rates by study arm were estimated using a multilevel mixed-effects model. Covariates were assessed via metaregression and presented as a forest plot. For each study, the risk of bias was assessed using the Cochrane Collaboration appraisal tool (RoB2) for randomized trials and randomized cross-over trials. Three main risk of bias (RoB) domains (selection and detection biases) were associated with the completion rate. These were included to explore potential biases in the reported information. In total, 62 trials comprising 122 study arms and 3523 participants were included. The overall pooled completion rate was 0.84 [95% confidence interval (CI): 0.80, 0.87] with lower rates of completion in trials published within the last 10 y. Low completion rates were associated with fecal sampling (0.75, 95% CI: 0.50, 1.00), study duration of 4–8 wk (0.79, 95% CI: 0.40, 1.00), and having a low RoB in the management of missing data (0.74, 95% CI: 0.34, 1.00). Overall, the completion rates of patients with IBD participating in controlled dietary intervention trials was high, and there was <15% variation in completion rates in relation to trial design. No linear correlation with trial duration was found. The most pronounced association with low completion was a comprehensive intervention content, i.e., fecal sampling.

This study was registered at PROSPERO CRD42022327783.

**Statement of significance**

When planning a randomized dietary trial that includes patients diagnosed with IBD, our findings suggested reconsidering the feasibility of a trial with comprehensive interventions. We suggested accounting for an additional 22% of participants in the sample size estimation and an additional 33% if planning fecal sampling.

## Introduction

Dietary intervention trials require many resources and are challenged by difficulties controlling participants’ dietary behavior according to controlled clinical trials standards. A frequent bias in health behavior change trials arises from differential attrition from the assigned intervention, resulting in selection bias and inadequately powered results or results favoring the control group [1]. Consequently, such trials risk low protocol adherence and high attrition rates, which may compromise study results and contribute to resource waste such as reduced power, compromised validity and generalizability [2,3]. Accounting for feasibility in the study design and planning phase of dietary research is a key step in improving research standards.

In inflammatory bowel disease (IBD), there is a growing demand for dietary recommendations to manage the disease course, and evaluation of diets for patients with IBD is a rapidly growing research area [4]. IBD is a chronic inflammation of the intestines and characterized by pain and rectal bleeding [5,6]. Despite many options for medical and biological therapies, many patients do not benefit from treatment, thus seek lifestyle changes to manage the disease course [5,6]. Observational studies report high motivation among patients with IBD adapting to dietary modifications that may alleviate their symptoms [7,8]. However, dietary intervention trials are often difficult to implement due to the strong preferences for individual dietary habits [9,10], and 2 systematic reviews evaluating the effects of dietary intervention trials on the disease course of IBD highlighted challenges with participant recruitment and retention as major limitations of their included trials [11,12].

Evidence-based research (metaresearch or research on research) seeks to identify best practices and evaluates reasons for systematic biases by taking a bird’s-eye perspective on the steps in the research process rather than criticizing individual studies [13,14]. The reasons behind attrition from dietary intervention studies in IBD due to trial methodology have not been reviewed systematically, but may contribute important insights for future trials and prevent research waste. Hence, this metaresearch study aimed to evaluate the completion rates of dietary intervention trials of patients with IBD and identify design features associated with the trial completion rate, with particular interest in trial duration.

## Methods

The study protocol was approved by all authors (Supplementary Material 1) and uploaded to the International Prospective Register of Systematic Reviews (PROSPERO) before conducting the literature search (CRD42022327783). Ethical approval was not required. The PRISMA 2020 checklist for reporting systematic reviews is presented in Supplementary Material 2.

### Study selection

We systematically searched MEDLINE (Ovid), Embase (Ovid) and CINAHL (EBSCO) databases on the 13 May, 2024 for randomized controlled trials (RCTs) studying any diet intervention for patients with IBD. Search blocks were defined based on a Patient/Population, Intervention, Comparison, and Outcome [15], designed research question and included study design (RCT), intervention type (diet), and study population [IBD, Crohn’s disease (CD), or ulcerative colitis (UC)]. Search terms for intervention type and population were inspired by Cochrane reviews [12,16], and quality checked by a research librarian (full search in Supplementary Material 1 as part of the study protocol). All citations were uploaded to Covidence, and duplicates were removed [17].

Studies were selected after reviewing titles and abstracts, followed by full-text screening. Two authors (LG and 1 of the 3: ZH, NR, and CM) independently reviewed titles, abstracts, and full texts with agreement required before progression and study inclusion. Disagreements were solved through discussion. If no consensus was achieved, inclusion was based on the majority vote.

### Eligibility criteria

Inclusion criteria were RCTs assessing a complete diet or macronutrient supplement compared with any other intervention, placebo, or habitual diet in patients with IBD. To prevent the inadvertent exclusion of dietary trials due to outcome-measure choices, studies were included regardless of which outcome they addressed. Trials of hospital-controlled meals or clinical nutrition supplements (e.g., parenteral nutrition) were excluded as the trial setting and dietary intervention of such trials were controlled by clinicians, differing from intervention trials controlled by patients at home. Language was restricted to Scandinavian languages, English and Spanish. No restrictions by publication date were applied. Cross-over trials were treated as a single study arm.

### Data collection and quality assessment

Two authors (LG and one of the 3: ZH, NR, and CM) extracted data from all included studies and evaluated the risk of bias (RoB) using the revised Cochrane Collaboration tools for assessing RoB in randomized trials and randomized cross-over trials [18]. Extracted data included study identification, participant baseline characteristics, population, intervention diet, comparator diet, study duration, intervention delivery, type of biologic samples collected, dietary compliance measures, and flow of participants by study arm, i.e., how many were randomized, analyzed, and not complying with the diet, and reasons for not completing the trial (lost-to-follow-up). Data were extracted separately for each study arm of the included trials. All studies were categorized with an overall “low,” “some concerns,” or “high” RoB based on the 5 domains; RoB arising from the randomization process, deviations from the intended interventions, missing outcome data, measurement of the outcome, and selection of the reported results. For cross-over trials, the tool incorporates a sixth domain assessing RoB arising from period and carryover effects. Studies with all domains categorized as low RoB were assessed with an overall low RoB, and studies with ≥1 domain categorized as “high” were assessed at high RoB.

### Assessment of participant completion rate

The effect measure was the share of randomized participants, i.e., the intention-to-treat population, following study protocol and having complete outcome data for the primary outcome at follow-up, i.e., those included in the primary analysis.

### Independent variables

Metaregression analyses were performed across all included study arms while having a random effect, adjusting for the trial from which it was sampled. The covariates were prespecified binary study design complexity variables and baseline participant characteristics outlined in the study protocol (Supplementary Material 1). Design variables included *1)* dietary regimen (additive compared with restrictive diet), *2)* study design (parallel compared with cross-over trials), *3)* study duration (<4 compared with ≥4 wk and as continuous scale), *4)* collection of feces, blood and urine samples, *5)* dietary instructions before participation, *6)* any type of nonmonetary motivation to adhere to the assigned diet, for example, telephone calls or regular surveys of adherence, and *7)* whether the diet content was supplied to the participants. An additional preplanned variable of monetary motivation for adherence was excluded from the analyses after data extraction because none of the trials reported using monetary motivation methods.

Additive diets were defined as diets that added dietary supplements and/or food items to a habitual diet, and restrictive diets were defined as diets that changed habitual eating habits by restricting specific dietary components or food items, potentially while also introducing specific food items (study protocol, Supplementary Material 1). When the participants’ baseline diet was not reported, a specific diet controlling dietary habit was categorized as restrictive. Any study arms receiving dietary guidance to comply with national health recommendations were also categorized as restrictive owing to the fact that guideline adherence is often inadequate [19].

Nonmonetary motivation strategies were defined as contacts between trial personnel and participants used to monitor dietary adherence, including face-to-face meetings, phone calls, and text-based communications.

Participant characteristic variables included sex, age, diagnosis (i.e., UC, CD, or mixed IBD diagnoses), and the share of participants using biologic therapy.

Post hoc, additional RoB variables reflecting internal validity [20], included randomization sequence blinded and concealed (selection bias), blinding of outcome assessors (detection bias), handling of missing outcome data (attrition bias), and blinding of participants. The analyses included these variables to assess possible confounding biases in the included trials.

## Data synthesis

Study characteristics were presented descriptively. A flowchart of the study selection process was adapted from the PRISMA guidelines [21]. All study arms were included in the overall synthesis of the study completion rate. The completion rate was estimated in a multilevel mixed effect model as prespecified in the study protocol (Supplementary Material 1) and presented by study arm for all included trials in a forest plot with an overall completion rate and 95% confidence intervals (CIs). We used a generalized linear mixed model approach, as proposed by Lin and Chu [22], due to the advantages of within-unit variance estimation and interpretability. Fixed effects were included for the design characteristics as a subgroup, whereas random effects were applied for the trial. The difference in completion rates between study arms was synthesized for parallel-designed trials and presented in a separate forest plot. In this plot, trials with >2 arms, the control group was proportionally divided across the active intervention arms. The difference in completion rates between participant characteristics was synthesized for trials including adult participants only.

The effect of the numeric variables was assessed using metaregression and presented as the independent completion rates and the risk ratio (RR) of completion between binary categories or per 1-unit increase for continuous variables. The statistical alpha level 0.05 was considered statistically significant, and 95% CI using the Wilson score method was estimated to address potential asymmetry and small samples. Heterogeneity was evaluated using τ^2^ across trials and *I*^2^ assessing the residual variance derived from the trials. We explored potential sources of heterogeneity by subgroup analyses and metaregression. The robustness of significant comparisons was evaluated using the *E*-value for the RR as a measure of potential residual confounding [23]. All statistical analyses were performed using the package *meta* in the software R Statistics version 4.4.2 developed by the R Core Team.

### Missing data in independent variables

When information about an independent variable was unavailable, the trial was not included in the data synthesis for that specific association. Data imputation testing the effect of missing data was not performed. In study arms with participant characteristics reported as 1 group rather than by study arm, the age of participants was assumed equal in all arms, and the number of females per study arm was estimated based on the total population and the size of each study arm. When an independent variable was not reported, the respective study arms were excluded from the synthesis.

### Sensitivity analyses

We included a sensitivity analysis of the study duration variable comparing trials of ≤4 wk to longer trials and additional assessments of study duration to evaluate our prespecified assumption of the linearity of this variable. Here, we compared trials with a duration of >4, ≤8, >8, ≤12 wk, and trials lasting longer than 12 wk to those lasting ≤4 wk. We further stratified the main results by publication date before 2010 or equal to or after 2010 corresponding to the updated CONSORT statement that required adoption of additional reporting standards by increased focus on transparent and unbiased reporting and expanded reporting items addressing trial design [24,25]. Due to 2 very trials having very high random weights in the assessment of differential attrition, a sensitivity analysis excluding these 2 trials was conducted. This showed very high weighting of additional 3 trials which were then excluded in a second sensitivity analysis.

## Results

**FIGURE 1:**
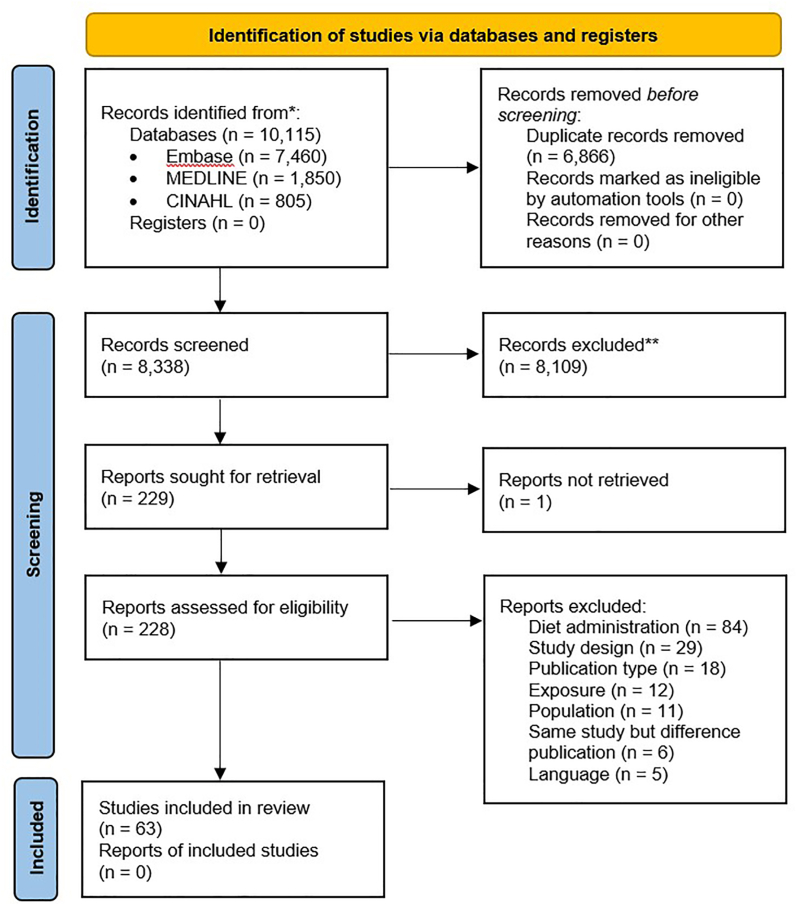
PRISMA flowchart [21] of the study selection process.

After deduplication, 8339 publications were screened, and 230 were selected for full-text assessment. Figure 1 illustrates the 63 unique dietary trials published between 1965 and 2024; 53 from developed and 10 from developing countries based on the United Nation’s 2024 list [26]. In total, 11 trials used a cross-over design, and 52 trials used a parallel design. One cross-over trial [27] reported the 2 study arms separately; hence, these were included as 2 separate study arms. All other cross-over trials were included, with 1 study arm per trial. Parallel trials were included with all their respective study arms. One of the parallel trials [28] assessed patients with both UC and CD but kept the diagnoses separate throughout the paper; hence, this study was included as a total of 4 study arms, i.e., 1 active group and 1 control group for each diagnosis. Participant flow was not available for 1 trial [29]. Thus, this trial was excluded from our analyses. In total, 62 trials comprising 122 study arms were analyzed.

### Trial characteristics

Trial characteristics are presented in Table 1 sorted by publication date within categories of the overall trial design and participant characteristics and in Supplementary Material 3 offering the option to sort by the other reported characteristics. Outcome measures and primary findings of the included trials are reported in Supplementary Material 4. In total, 20 trials (40 study arms) assessed patients with CD, 32 trials (62 study arms) assessed patients with UC, and 10 trials (20 study arms) assessed mixed diagnoses. Six trials (11 study arms) assessed children aged <18 y, whereas all others assessed adult participants. The intervention diet was a complete diet and/or selected food items in 39 trials (78 study arms), and a macronutrient supplement such as oil, drink, or powder in 23 trials (44 study arms).

**TABLE 1:** Characteristics of included trials and study arms grouped by study design

Study ID	Country	Recruitment period	Trial duration (wk)^1^	Population	Study arm	Diet content	Diet type
Trials with adult participants grouped by study design and diagnose(s) and sorted by publication date							
Parallel-designed trials							
**UC participants**							
Alborzi Avanaki 2024 [30]	Iran	Not reported	6	38 adults with UC	1	GFD + gluten-free bread	Mixed
					2	GFD + bread with gluten	Mixed
Kedia 2024 [31]	India	Sep/2019–Mar/2020 + Aug/2020–Apr/2022	8	97 adults with UC	1	Placebo water	Additive
					2	Coconut water	Additive
Lluansi 2024 [32]	Spain	Dec/2019 – Aug/2021	8	31 adults with UC	1	Sourdough bread	Additive
					2	Placebo bread	Additive
Haskey 2023 [33]	Canada	Apr/2017–Sep/2021	12	32 adults with UC	1	Mediterranean	Restrictive
					2	Standard Canadian diet	Restrictive
Miyaguchi 2023 [34]	Japan	Aug/2018–Mar/2021	24	20 adults with UC	1	Zink + omega-3 diet	Additive
					2	Habitual diet	None
Morshedzadeh 2023 [35]	Iran	Sep/2018–Jun/2019	12	70 adults with UC	1	Flaxseed powder	Additive
					2	Habitual	None
Kedia 2022 [36]	India	Sep/2019–Mar/2020	8 48^2^	73 adults with UC	1	Anti-inflammatory diet	Restrictive
					2	Habitual	None
Keshteli 2022 [37]	Canada	2014–2017	26	53 adults with UC	1	Anti-inflammatory diet	Restrictive
					2	Standard Canadian diet	Restrictive
Sarbagili Shabat 2022 [38]	Italy/Israel	May/2017–Dec/2020	12	51 adults with UC	1	No diet	None
					2	UC exclusion diet + FT	Restrictive
					3	UC exclusion diet	Restrictive
Nyman 2020 [39]	Sweden	2-y period	24	130 adults with UC	1	Oat bran	Additive
					2	Wheat bran	Additive
Bamba 2018 [40]	Japan	Dec/2015–Feb/2017	16 8	11 adults with UC	1	Fermented vegetable beverage	Additive
					2	Fermented vegetable beverage	Additive
Jian 2018 [41]	China	Apr/2012–Sep/2015	26	97 adults with UC	1	IgG exclusion diet	Restrictive
					2	Habitual	None
Hvas 2016 [42]	Denmark	Mar/2012–Sep/2014	8 4	24 adults with UC	1	Flavored whey protein	Additive
					2	No diet	None
Kyaw 2014 [43]	United Kingdom	Not reported	24	130 adults with UC	1	Low carbohydrate, low fat, high protein	Restrictive
					2	Habitual	None
Faghfoori 2011 [44]	Iran	Not reported	8	41 adults with UC	1	Germinated barley foodstuff	Additive
					2	No diet	None
Casellas 2007 [45]	Spain	Not reported	2	19 adults with UC	1	Inulin	Additive
					2	Matodextrin	Additive
Seidner 2005 [46]	United States	Sep/1994–Dec/1997	26	121 adults with UC	1	UC nutritional supplement	Additive
					2	Carbohydrate drink (placebo)	Additive
Kato 2004 [47]	Japan	Sep/2001–Mar/2003	12	20 adults with UC	1	Fermented milk	Additive
					2	Placebo milk	Additive
Ishikawa 2003 [48]	Japan	Apr/1998–Sep/1998	52	21 adults with UC	1	Fermented milk	Additive
					2	No diet	Additive
Kanauchi 2002 [49]	Japan	Not reported	4	18 adults with UC	1	Germinated barley foodstuff	Additive
					2	No diet	None
Fernandez-Banares 1999 [50]	Spain	Jan/1993–May/1996	52	105 adults with UC	1	HUSK seeds	Additive
					2	No diet + treatment	None
					3	HUSK + treatment	Additive
Almallah 1998 [51]	United Kingdom	Not reported	26	18 adults with UC	1	Fish oil	additive
					2	Sunflower oil	additive
Candy 1995 [52]	South Africa	Not reported	6	18 adults with UC	1	Elimination diet	Restrictive
					2	Habitual	None
Hawthorne 1992 [53]	United Kingdom	Not reported	52	96 adultswith UC	1	Fish oil	Additive
					2	Olive oil (placebo)	Additive
Wright 1965 [54]	United Kingdom	Not reported	52	77 adults with UC	1	Milk-free	Restrictive
					2	Milk-free + GFD	Restrictive
					3	Habitual	None

CD participants							
Lewis 2021 [55]	United States	Sep/2017–Oct/2019	12	197 adults with CD	1	Mediterranean	Restrictive
					2	SCD	Restrictive
Albenberg 2019 [56]	United States	Nov/2013–Jun/2015	49	213 adultswith CD	1	High meat	Restrictive
					2	Low meat	Restrictive
Gunasekeera 2016 [57]	United Kingdom	Jul/2007–Sep/2010	4	98 adults with CD	1	IgG exclusion diet	Restrictive
					2	Sham exclusion diet	Restrictive
Machado 2015 [58]	Brazil	Feb/2012–Oct/2012	16	68 adults with CD	1	Whey protein	Additive
					2	Soy protein	Additive
Brotherton 2014 [59]	United States	Not reported	4	7 adults with CD	1	High fiber and low refined carbohydrates	Restrictive
					2	Sham diet	Restrictive
Benjamin 2012 [60]	India	Nov/2005–Nov/2008	8	30 adults with CD	1	Glutamine	Additive
					2	Whey protein	Additive
Benjamin 2011 [61]	United Kingdom	Sep/2006–Apr/2009	5 4	103 adults with CD	1	Fermented oligosaccharides	Additive
					2	Maltodextrin	Additive
Bartel 2008 [62]	Austria	Jan/2004–Jan/2004	24 6	18 adults with CD	1	High meat/high fat	Restrictive
					2	No meat, low fat, high carbohydrate	Restrictive
Eivindson 2005 [63]	Denmark	Not reported	9	31 adults with CD	1	Omega-3	additive
					2	Omega-6	additive
Lomer 2005 [64]	United Kingdom	Dec/1999–Jan/2002	16	83 adults with CD	1	Low particle diet	Restrictive
					2	Normal particle diet	Restrictive
					3	Low particle diet + calcium supplement	Restrictive
					4	Normal particle diet + calcium supplement	Restrictive
Nielsen 2005 [65]	Denmark	Not reported	9	31 adults with CD	1	Omega-3	Additive
					2	Omega-6	Additive
Lomer 2001 [66]	United Kingdom	Not reported	16	20 adults with CD	1	Micro particle diet	Restrictive
					2	Sham dietary advice	Restrictive
Den Hond 1999 [67]	Belgium	Not reported	4	14 adults with CD	1	Glutamine	Additive
					2	Glycine (placebo)	Additive
Ritchie 1987 [68]	United Kingdom	Sep/1980–Aug 1983	104	352 adults with CD	1	Refined carbohydrates	Restrictive
					2	Unrefined carbohydrates	Restrictive
Levenstein 1985 [69]	Italy	Mar/1981–Dec/1981	130	71 adults with CD	1	Low fiber	Restrictive
					2	Standard fiber	Additive

Mixed IBD participants							
Liso 2022 [70]	Italy	Sep/2019–Dec/2020	8	47 adults with IBD	1	Red juice (placebo)	Additive
					2	Purple corn extract	Additive
Lacerda 2021 [71]	Portugal	Not reported	8	25 adults with IBD	1	Mediterranean diet + functional foods	Restrictive
					2	Mediterranean diet	Restrictive
Cox 2020 [72]	United Kingdom	Feb/2016–May/2017	4	52 adults with IBD	1	Low FODMAP	Restrictive
					2	Exclusion diet (sham)	Restrictive
Bodini 2019 [29]	Italy	Not reported	6	55 adults with IBD	1	Low FODMAP	Restrictive
					2	Standard FODMAP diet	Additive
Yilmaz 2019 [28]	Turkey	May/2015–Dec/2016	4	45 adults with IBD	1	Kefir Colitis-group	Additive
					3	Habitual Colitis-group	None
					2	Kefir Crohns-group	Additive
					4	Habitual Crohns-group	None
Pedersen 2017 [73]	Denmark	Jun/2012–Nov/2013	6	89 adults with IBD	1	Low FODMAP	Restrictive
					2	Habitual	None
Brunborg 2008 [74]	Norway	Apr/2003–Nov/2003	2	45 adults with IBD	1	Seal oil	Additive
					2	Cod liver oil	Additive
Bjorck 2000 [75]	Sweden	Not reported	8 4	53 adults with IBD	1	Hydrothermally processed cereal	Additive
					2	Ordinary cereal	Additive

Cross-over designed trials							
UC participants							
Laatikainen 2023 [76]	Finland	2021–2022	4 wk 2 wk	7 adults with UC	1–2	Carrageenan product vs. beta-glucan	Additive
Melgaard 2022 [77]	Denmark	Jul/2018–Aug/2020	8 wk	19 adults with UC	1–2	Low FODMAP + provocation foods	Mixed
Fritsch 2021 [78]	United States	Feb/2015–Sep/2018	10 wk 8 wk	26 adults with UC	1–2	Low fat/high fiber vs. standard American diet	Restrictive
Morvaridi 2020 [27]	Iran	Jan/2018–Aug/2018	54 d 40 d	40 adults with UC	1	Placebo vs. olive oil	Additive
					2	Olive oil vs. placebo	Additive

CD participants							
Halmos 2016 [79]	Australia	Mar/2009–May/2011	10 wk 6 wk	9 adults with CD	1–2	Low FODMAP vs. sham diet	Restrictive
James 2015 [80]	Australia	Jun/2002–Oct/2008	48 d 34 d	25 adults with UC	1–2	Resistant starch vs. wheat bran fiber	Additive
Walters 2014 [81]	United States	Not reported	13 wk 8 wk	6 adults with CD	1–2	SCD vs. low-residue diet	Restrictive
Bentz 2010 [82]	Switzerland	Not reported	12 wk 6 wk	40 adults with CD	1–2	IgG exclusion diet vs.sham exclusion diet	Restrictive

Mixed IBD participants							
Cox 2017 [83]	United Kingdom	Mar/2014–Oct/2015	4 wk 12 d	32 adults with IBD	1–4	Low FODMAP + provocation foods	Mixed

Trials with child participants grouped by study design and sorted by publication date							
Parallel designed trials							
Allen 2022 [84]	United Kingdom	Not reported	6	23 children with CD	1	BC milk	Additive
					2	Placebo milk	Additive
El Amrousy 2022 [85]	Egypt	Apr/2020–Apr/2021	12	100 children with IBD	1	Mediterranean	Restrictive
					2	Habitual	None
Suskind 2020 [86]	United States	Nov/2015–Dec/2018	12	16 children with CD	1	SCD	Restrictive
					2	SCD + oats and rice	Restrictive
					3	Whole food diet	Restrictive
Strisciuglio 2013 [87]	Italy	Not reported	52	29 children with UC	1	Cow’s milk protein elimination diet	Restrictive
					2	Habitual	None

Cross-over designed trials							
Kaplan 2022 [88]	United States	Apr/2018–Dec/2019	34 32	54 children with IBD	1–2	SCD vs. modified SCD	Restrictive
Ejderhamn 1992 [89]	Sweden	Not reported	182 156	12 children with UC	1–2	Wheat fiber vs. isphagula	Additive

Abbreviations: CD, Crohn’s disease; FODMAP, fermentable oligosaccharides, disaccharides, monosaccharides and polyols; FT, fecal transplantation; GFD, gluten-free diet; IBD, inflammatory bowel disease; ID, identity document; SCD, specific carbohydrate diet; UC, ulcerative colitis.

1 Diet duration shown in italics if diet duration differs from study duration.

2 Maintenance diet continued after the primary scope of the study.

### Trial design variables

Trial duration ranged from 2 wk to 3.5 y with a median duration of 12 wk (IQR: 6–24). Five (4%) study arms were shorter than 4 wk. In total, 55 (45%) study arms were categorized as additive, and 46 (38%) were restrictive. Four (3%) study arms from 3 different trials [30,77,83] were categorized as mixed dietary regimens due to a combination of a restrictive diet along with additional provocation foods, and 17 (14%) study arms were defined as habitual diet. Only 1 study arm from a single trial [60] reported baseline dietary intake showing an altered intake due to the intervention diet. Therefore, this and all other study arms receiving dietary guidance aiming at compliance with national recommendations or specific diets, such as the anti-inflammatory diet or the Mediterranean diet, were categorized as restrictive. Two (4%) of the additive study arms from 1 trial [60] supplied all energy needs for the participants. Six (13%) of the restrictive study arms from 4 trials [55,71,78,79] supplied all meals adjusted for individual energy intake, and 2 (4%) of the restrictive study arms from 1 trial [90] supplied all meals and did not adjust for individual energy needs.

Fecal sampling was included in 71 (58%) study arms, blood sampling in 98 (80%) study arms, and urine sampling in 10 (8%) study arms. Dietary guidance was provided by a study dietitian in 47 (39%) study arms, a nondietary or nonspecified trial associate in 38 (31%) study arms, and no instructions for the intervention diet were described in 37 (30%) study arms. Furthermore, the diet or supplement was fully or partially provided to participants in 77 (63%) study arms, participants were blinded to the intervention in 63 (52%) study arms, and nonmonetary motivation methods for retaining dietary adherence were used in 75 (61%) study arms.

### Participant characteristics variables

The median (IQR) proportion of females was 53% (44%–63%) in 113 (93%) study arms reporting sex of participants. Age of participants was reported as median (IQR) in 50 (41%) study arms having an overall median (IQR) age of 38.0 (35.2–43.0) y, and as mean (SD) in 72 (59%) study arms having an overall median (IQR) age of 37.9 (33.9–40.9) y. For 2 parallel-designed trials [71,86], age and sex were estimated based on the entire study population. Disease activity was mild to moderate in all 99 (81%) study arms reporting disease activity. Due to limited published information, disease activity was excluded from the data synthesis. Biologic treatment was reported in 43 (35%) study arms, with 0%–100% of participants using biologic treatment during the trial.

### Overall completion rate

**FIGURE 2:**
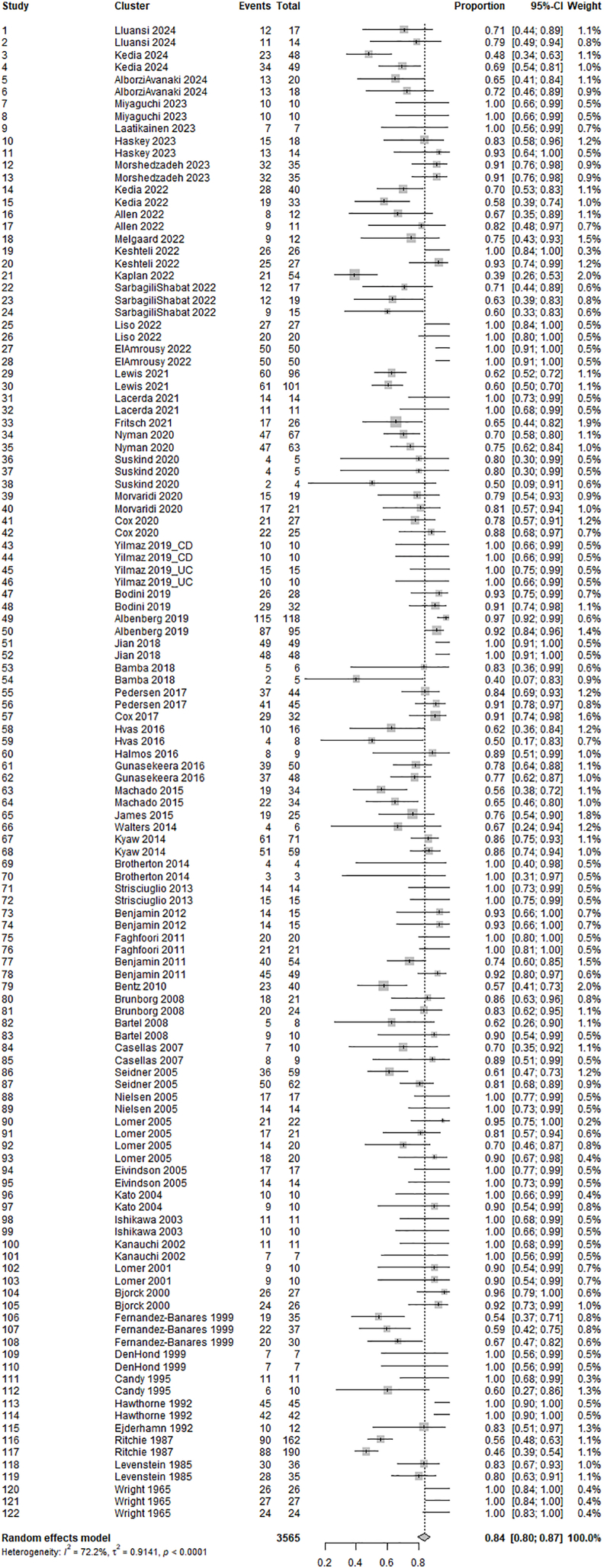
Forest plot of overall completion rate by study arm for the intercept-only model including child and adult trials. Fixed effects were included for the design characteristics as a subgroup, whereas random effects were applied for the trial. CD, Crohn’s disease; CI, confidence interval; RR, risk ratio; UC, ulcerative colitis.

**FIGURE 3:**
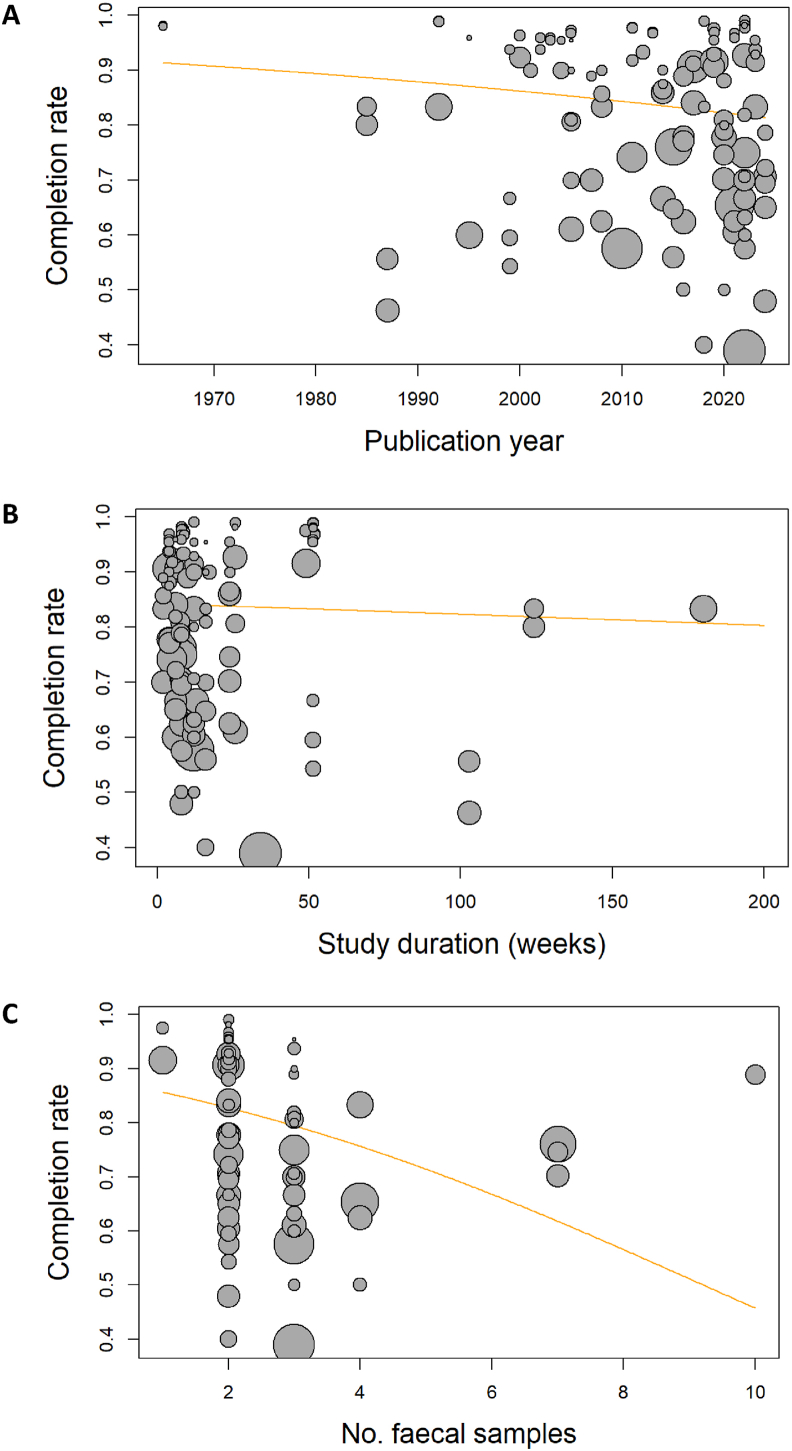
Bubble plot of completion rate according to (A) publication year, (B) study duration measured in weeks, and (C) the number of fecal samples collected during the trial, along with the corresponding metaregression line (orange). The size of the bubbles represents the sample size of each study arm.

In total, 3523 participants were included in the synthesis, and 2720 (77%) completed the trials. As illustrated in Figure 2, the proportion of participants completing the individual study arms ranged from 39% to 100%, with a pooled completion rate of 0.84 (95% CI: 0.80, 0.87). Full completion with no protocol violations was reported in 38 (31%) of the study arms. The completion rate in trials published 10 y before the search date was 6% (95% CI: 1%, 11%, *P* = 0.010, *E* = 1.33) lower than in older trials (Figure 3A).

### Association between completion rate and independent variables

Results of the preplanned stratified meta-analyses are presented in TABLE 2, TABLE 3, and the additional analyses of different study duration categories are presented in Table 4. Overall, the completion rates within each assessed category varied <15% from the overall completion rate. The highest rate was observed in trials without fecal sampling, whereas the lowest rate was seen in cross-over designs.

**TABLE 2:** Results of the stratified meta-analysis for design characteristics modifying completion rate (univariate analysis) across all child and adult trials

Analysis	Arms (*n*) (trials)	Meta-analysis						
		Completion rate	95% CI		*τ*^2^	*I*^2^ (%)	RR (95% CI)	*P* value for association
Overall	122 (62)	0.84	0.80	0.87	0.91	72	—	
Diet regimen^1^	—	—	—	—	0.82	72	0.97 (0.91, 1.03)	0.15
Restrictive	47 (29)	0.82	0.42	1.00	—	—		
Additive	54 (33)	0.84	0.48	1.00	—	—		
Study design	—	—	—	—	0.83	72	1.18 (1.12, 1.24)	<0.001^4^
Parallel	110 (52)	0.86	0.54	1.00	—	—		(*E* = 1.64)
Cross-over	12 (11)	0.72	0.20	1.00	—	—		
Study duration (wk)	—	—	—	—	0.84	72	0.99 (0.92, 1.05)	0.37
<4	5 (3)	0.83	0.19	1.00	—	—		
≥4	117 (59)	0.84	0. 5	1.00	—	—		
Fecal samples	—	—	—	—	0.81	72	0.87 (0.81, 0.92)	<0.001^4^ (*E* = 1.58)
Yes	73 (38)	0.75	0.50	1.00	—	—		
No	49 (24)	0.91	0.39	1.00	—	—		
Blood samples	—	—	—	—	0.84	72	1.06 (1.00, 1.12)	0.030^4^
Yes	98 (48)	0.85	0.53	1.00	—	—		(*E* = 1.30)
No	24 (14)	0.80	0.26	1.00	—	—		
Urine samples	—	—	—	—	0.84	72	1.06 (0.95, 1.16)	0.15
Yes	10 (5)	0.88	0.00	1.00	—	—		
No	112 (57)	0.83	0.55	1.00	—	—		
Diet provided	—	—	—	—	0.84	72	0.95 (0.94, 1.01)	0.06
All/some	81 (45)	0.82	0.53	1.00	—	—		
None	41 (27)	0.86	0.29	1.00	—	—		
Instructions^2^	—	—	—	—	0.83	72	0.99 (0.93, 1.04)	0.31
By dietician	87 (25)	0.84	0.50	1.00	—	—		
None	35 (18)	0.85	0.35	1.00	—	—		
Motivation^3^	—	—	—	—	0.80	69	0.94 (0.89, 0.99)	0.006^4^
Yes	77 (38)	0.81	0.46	1.00	—	—		(*E* = 1.33)
No/unclear	45 (24)	0.87	0.47	1.00	—	—		
Randomization	—	—	—	—	0.83	71	0.92 (0.87, 0.97)	<0.001^4^
Low RoB	66 (34)	0.81	0.47	1.00	—	—		(*E* = 1.40)
Some RoB	60 (28)	0.88	0.41	1.00	—	—		
Blinding of outcome assessors	—	—	—	—	0.82	71	0.90 (0.85, 0.95)	<0.001^4^ (*E* = 1.46)
Low RoB	62 (32)	0.79	0.50	1.00	—	—		
Some/High RoB	60 (30)	0.88	0.41	1.00	—	—		
Handling of missing data	—	—	—	—	0.82	72	0.86 (0.81, 0.91)	<0.001^4^ (*E* = 1.60)
Low RoB	25 (13)	0.74	0.34	1.00	—	—		
Some/high RoB	97 (49)	0.86	0.53	1.00	—	—		
Blinding of participants	—	—	—	—	0.84	72	0.94 (0.89, 0.99)	0.010^4^ (*E* = 1.32)
Yes	61 (34)	0.81	0.53	1.00	—	—	—	
No/unclear	61 (28)	0.86	0.38	1.00	—	—	—	

Fixed effects for trial arms while a random factor for the specific trial. Variables below the line are domains of risk of bias on the internal validity.

Abbreviations: CI, confidence interval; RoB, risk of bias; RR, risk ratio.

1 Restrictive diets were defined as those changing habitual diet by restricting specific dietary components or food items, and additive diets were defined as those adding dietary supplements and/or food items to habitual diet.

2 Diet instructions by other than dieticians/nutritionists excluded.

3 Any nonmonetary method used to encourage protocol compliance during participation.

4 Significant level < 0.05.

**TABLE 3:** Results of the metaregression for adult participant baseline characteristics modifying completion rate (univariate analysis)

Analysis	Study arms (*n*) (trials)	Meta-analysis				
		τ^2^	*I*^2^	RR^1^	95% CI	*P* value for association
Female (%)	101 (52)	0.70	95 %	1.21	0.34, 4.33	0.76
Age (y)	96 (49)	0.71	95 %	0.97	0.93–1.01	0.20
UC diagnose (%)	103 (51)	0.77	95 %	0.98	0.53–1.82	0.95
Biologic therapy (%)	40 (21)	0.55	100 %	1.58	0.34–7.38	0.56

Fixed effects for trial arms while random effects for the specific trial.

Abbreviations: CI, confidence interval; RR, risk ratio; UC, ulcerative colitis.

1 Relative to a 1-unit increase.

**TABLE 4:** Results of the stratified meta-analysis for explorative study duration measures modifying completion rate (univariate analysis)

Analysis	Arms (*n)*	Meta-analysis						
		Completion rate	95% CI		*τ*^2^	*I*^2^ (%)	RR (95% CI)^1^	*P* value for association
Study duration (wk)								
≤4	20	0.85	0.48	1.00	—	—	—	
>4	102	0.83	0.51	1.00	0.82	71	1.02 (0.97, 1.06)	0.21
>4 to ≤8	30	0.79	0.40	1.00	0.76	68	1.07 (0.99, 1.15)	0.08
>8 to ≤12	27	0.86	0.18	1.00	0.80	67	0.98 (0.86, 1.10)	0.36
>12	45	0.84	0.29	1.00	0.83	72	1.01 (0.92, 1.09)	0.45

Fixed effects for trial arms while a random factor for the specific trial.

Abbreviations: CI, confidence interval; RR, risk ratio.

1 Comparing with studies ≤4 wk.

No linear effect of study duration on completion rate was found [effect estimate = –0.1% (–0.3% to 0.5%) per week, *P* = 0.55, Figure 3B]. The sensitivity analyses revealed a potential nonlinear association between completion and study duration as completion in trials lasting 4–8 wk tended to be lower (7%; 95% CI: 1% higher, 15% lower) compared with trials of ≤4 wk duration, whereas no differences were found between the short trials (≤4 wk) and those lasting 8–12 or >12 wk (Table 4).

Compared with the overall estimate, the completion rate was lower in trials collecting fecal samples (75%; 95% CI: 50%, 100%) and higher in trials without fecal sampling (91%; 95% CI: 39%, 100%). The difference within this variable was 13% (95% CI: 8%, 19%, *P* < 0.001, *E* = 1.58). Post hoc exploration indicated that increasing the number of fecal samplings was correlated with reduced trial completion. Each additional sampling corresponded to a 20% reduction in completion rate (95% CI: 7%, 30%, *P* = 0.003, *E* = 1.79, Figure 3C).

**FIGURE 4:**
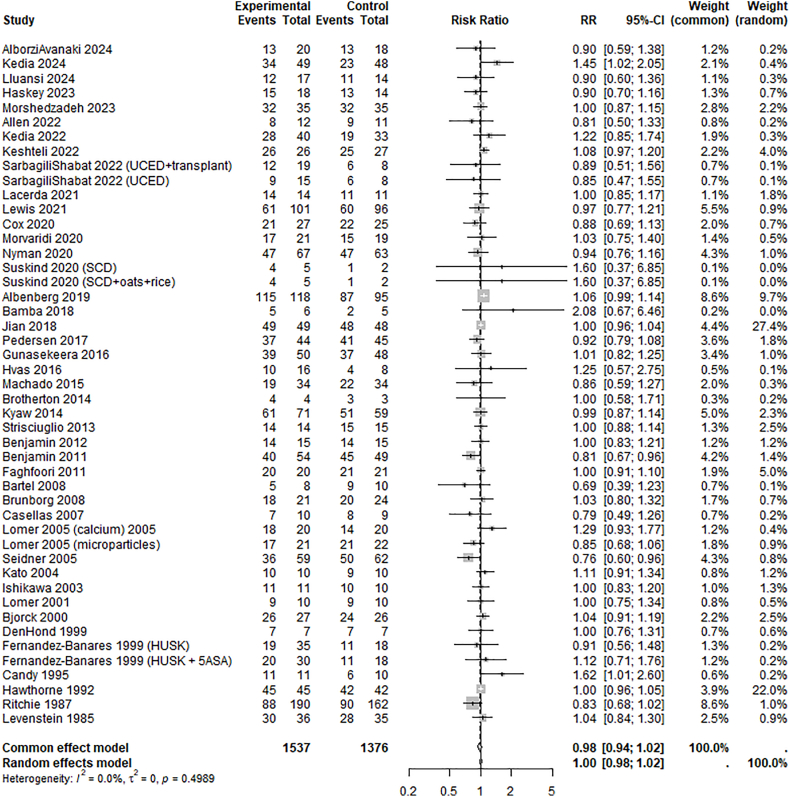
Forest plot of the relative risk of completion between study arms per trial, having a parallel study design. RR>1 represents larger completion rates in the experimental arm; RR<1 represents larger completion rates in the control arm. In 3 trials with 2 experimental arms compared with 1 control arm, the control arm was split between the experimental arms. CI, confidence interval; RR, risk ratio.

In cross-over designed trials, the completion rate was lower than the overall estimate (72%; 95% CI: 20%, 100%) and 18% (95% CI: 12%, 24%, *P* < 0.001, *E* = 1.64) lower than in parallel-designed trials. In trials not employing nonmonetary motivation strategies, the completion rate was slightly higher than the overall estimate (81%; 95% CI: 46%, 100%) and 6% (95% CI: 1%, 11%, *P* = 0.006, *E* = 1.33) higher than in trials employing such strategies. All other trial design and population variables were not associated with completion rate (TABLE 2, TABLE 3). Figure 4 illustrates the RR for trial completion between the study arms in the parallel-designed trials. The corresponding sensitivity analyses excluding trials with high random weighting are presented in Supplementary Material 8.

### Associations between completion rate and RoB

Summary measures of the RoB assessment are presented in Supplementary Material 5. In total, 34 (55%) trials had low RoB in the randomization process, 32 (52%) in the blinding of outcome assessors, and 13 (21%) in the handling of missing outcome data of which 11 (85%) were published after 2010. The ITT population was reported in 35 trials of which 26 were published after 2010 (data not shown). Compared with the overall estimate, our post hoc analyses showed lower completion rates of trials with a low RoB in the randomization process (selection bias), blinding of outcome assessors (performance and detection bias), and handling of missing data (selection bias), with the latter being the most pronounced association of the RoB variables with 12% lower rate (Table 2). For all 3 RoB variables, the completion rate was slightly higher in trials having some or high RoB compared with the overall estimate (Table 2).

### Results stratified by publication date

Findings from the studies published since 2010 are reported in Supplementary Material 6, and findings from studies published before 2010 are reported in Supplementary Material 7. The overall completion rate in trials published before 2010 was 86% (95% CI: 79%, 91%) and 5% higher than in trials published since 2010, which had an overall completion rate of 82% (95% CI: 77%, 87%). In trials published before 2010, the within variable variance was smaller compared with trials published since 2010 for all variables except urine sampling. In both subsamples, the findings replicated those of the main findings (table) with only a few exceptions. A linear association between trial completion and an increasing number of stool sampling was found only in trials published since 2010. In the most recent trials providing some or all dietary items to the participants, the completion rate was lower than the overall estimate (78%; 95% CI: 0%, 100%). Also in the most recent trials, the completion rate was also lower than the overall estimate when keeping the participants blinded to the diet (75%; 95% CI: 0%, 100%). This estimate was 13% (95% CI: 6%, 20%, *P* < 0.001, *E* = 1.57) lower than in trials not doing so. The opposite trend was found in trials published before 2010 (no statistically significant difference within this variable was found in trials published before 2010). For the urine sampling, the 2 subsamples showed trends in the opposite direction of the primary, likely due to skewed and very small sample sizes.

## Discussion

This metaresearch study evaluated the completion rates of dietary intervention trials that included patients with IBD and assessed associations between trial completion and design features. The study found an overall high completion rate, yet lower than the completion rates reported in medical intervention trials in IBD [91]. The observed overall completion rates and low variance in completion associated with trial design features are in accordance with those of review papers evaluating randomized trials of health behavior changes, including dietary or physical activity interventions in various chronic diseases [1,92,93]. Nevertheless, an overall higher completion rate was found than in a meta-analysis of 6 randomized dietary intervention trials of patients with rheumatic arthritis [94]. Here, the lowest rates were reported in the active intervention groups compared with those following their ordinary diet [94]. These discrepancies with our study indicate that our findings are specific to both the research field of diet interventions and the patient group evaluated.

### Trial design variables reducing completion rates

Despite no apparent association found between trial duration and completion, our post hoc analyses of study duration indicated a nonlinear correlation between study duration and trial completion. For trials lasting ≤8 wk, our findings align with other dietary and health behavior change trials in other chronic diseases showing higher attrition in longer trials [1,93]. However, for the longer trials, this correlation could not be applied, which may indicate an overall lower participant burden in trials lasting >8 wk.

The findings of fecal sampling being associated with low trial completion are likely highly specific to patients with IBD, as fecal analyses are frequently part of this population’s medical examinations. Despite the lower completion rates, the difference from the overall rate was <15%, which in other populations may be much higher. Still, this finding contrasts with a recent survey of attitudes toward fecal sampling among 660 patients with IBD, reporting a high willingness to participate in fecal sampling trials [95]. However, this survey study may be skewed toward more positive responses, as less than half of the initial population responded. The study further highlighted that clear explanations of sampling purpose and procedure would increase successful participation [95]. Detailed information about the sampling procedure and motivation was poorly described in the published material of our included trials, and is outside the scope of this meta-analysis. Despite the limited variations from the overall completion rate, our findings of lower completion in trials employing fecal sampling or having a cross-over study design indicate that a more comprehensive intervention content may cause some participants to leave a trial early. Although the cross-over design variable may be biased by the skewed sample sizes, similar trends of lower completion in association with more complex interventions are also reported from dietary interventions in other chronic diseases [1].

Using nonmonetary motivation strategies for dietary adherence did not appear to improve trial completion, but rather the absence of such strategies. Given the considerable heterogeneity in how the trials were conducted, we did not pursue further analysis of this variable. This finding may reflect a strong interaction with other design features, as more complex diet interventions may correlate with higher efforts to maintain the participants’ engagement in the trial, whereas less complex interventions may not include this feature. Also, different methods of motivating participants (e.g., in-person or online) were not distinguished. On the basis of the included trials, we could not support previous findings of higher completion in association with monetary compensation for participation [96].

### Participant characteristic variables did not correlate with trial completion

Our findings regarding participant characteristics were based on limited published data and a large 95% CI, so no reliable conclusions can be drawn. Assessment of any detailed demographic features was beyond the scope of this study, as such data are seldom reported. In other areas, females have been reported to have higher adherence with weight-loss dietary interventions or general dietary recommendations than males [97,98]. Thus, a similar trend may apply to dietary interventions in the disease management of IBD. Sociodemographic factors such as smoking or high BMI were not assessed in this study but have previously been associated with adherence to a long-term weight-loss diet among participants with obesity [99]. In agreement, 2 extensive European population studies reported that those with the lowest level of education were less likely to adhere to healthy dietary recommendations [100,101]. Moreover, socioenvironmental factors have been identified as barriers to adherence with time-restricted eating interventions [92]. Together, these studies indicate that participant characteristics and cultural settings may be of relevance to completion rates.

### Different completion rates between publication periods

Despite legislative changes increasing the awareness of dietary health impacts and paradigm shifts in dietary research from researcher-centric trials toward patient-friendly designs and patient involvement in research, the overall completion rate for trials published before and after the CONSORT update in 2010 differed only minimally. The larger difference in completion rates between trials published before or after 2014 found in the primary analyses indicates that a potential shift in methodological approaches is more recent than the 2010 CONSORT update. This aligns with the recent increased emphasis on patient-relevant outcomes and public involvement in research since mid-2010s [102,103]. However, for trials published since 2010, a lower completion rate was found with the provision of some or all dietary items and with keeping participants blinded to the intervention content. These 2 associations were not present in trials published before 2010, which exemplifies the paradigm shift in dietary research that has influenced research management and participant behavior.

### RoB was associated with completion rates

The RoB variables reflect transparent reporting of trial conduct, participant flow, and data management. The findings related to these variables likely reflect potential reporting biases. The share of included trials analyzing the ITT population tripled after publication of the updated CONSORT guidelines in 2010 [24,25], and the share of trials with a low RoB in the management of missing data increased accordingly. The correlation between low completion rates and trials with low RoB in the handling of missing data most likely reflects increasingly transparent reporting rather than a true correlation with trial completion. Our findings of nonmonetary motivation strategies contradict other findings [95,96,[104], [105], [106]], but may also align with RoB in reporting participant flow, though such a correlation was not evaluated.

### Implications for dietary intervention effect estimates

This study did not assess the effect of attrition on study results or effect estimates. In other related fields, lack of or unclear blinding of participants and trial personnel (i.e., detection bias) has been associated with an exaggeration of treatment effects in RCTs, especially when studying subjective outcomes [107]. Despite no differential attrition in our included trials, the high proportion of trials at risk of detection bias may have inflated the reported findings in the trials, highlighting the need for better trial management. Moreover, a meta-epidemiological research study of 393 randomized controlled physical therapy trials across various diseases reported that inappropriate control of incomplete outcome data (i.e., bias due to missing outcome data) compared with an adequate use of the intention-to-treat principle tended to underestimate the treatment effect [108]. Similarly, the high proportion of dietary trials that did perform ITT analyses may have overestimated the effect of the diet intervention.

Our findings suggest accounting for an increased attrition in more complex dietary intervention trials, even in IBD, despite this population’s high motivation for disease management through dietary modifications [7,8]. An overall completion rate of 84% suggests a need to recruit an additional 19% of participants, whereas a completion rate of 75% in the trials employing fecal sampling suggests would need recruitment of an additional 33% participants. Still, this does not mitigate the risk of differential attrition (selective dropout) and selection bias in the effect estimates. Furthermore, assessments of good dietary trial management, such as targeted strategies for recruitment and retention and considerations of feasibility of the intended intervention, are required to assess methods for limiting participant attrition.

### Strengths and limitations

Our large sample size of randomized controlled dietary trials with patients with IBD due to very limited exclusion criteria highlights the strengths of this meta-epidemiological study. Moreover, the E-values for significant associations were high, indicating that these associations were strong. Still, our findings are based on data from dietary trials in IBD. Therefore, they may not be applicable in other conditions or intervention types due to different motives and diet tolerability between patients with chronic inflammation in the intestines compared with other populations. Moreover, this study is based on observational data extracted from publications and possible confounding by e.g., resources, expertise, and local research policies may exist [109]. Due to inconsistent reporting of participant characteristics, possible confounding may further arise from comorbidities, symptom burden, socioeconomic status, or sociodemographic factors. Different associations may further exist between child and adult participants, but associations between trial completion and participant characteristics of child participants were not evaluated due to the very small sample of trials including child participants. Also, due to the nature of our study, our findings may be subject to findings by chance [110] and should be interpreted as hypothesis-generating.

All findings are further subject to high variance and high heterogeneity arising from the varying trial designs as shown in Figure 2. This strongly indicates that the assessed trials share only limited characteristics. Although subgroup analyses would enable more homogeneous samples and stratified analyses may provide valuable insight into potential interaction between some of the assessed variables, comparisons with very low heterogeneity also compromise the generalizability within the field of interventional dietary research in IBD. Such stratified analyses were beyond the scope of the present metaresearch study and were not conducted. We also acknowledge that the binary comparisons may have left out important information and potentially led to an overestimation of the associations [111]. Also, we did not collect information on the number of outcomes reported by the trials, the intensity of intervention delivery, the motivation incentives, or the degree of control over energy intake, which could have provided more nuanced perspectives on trial completion as also indicated by previous reviews [1,96]. Moreover, the lower completion rate in trials published after 2010 and in trials with low RoB for internal validity suggests some confounding from reporting biases.

In conclusion, we found an overall completion rate of dietary intervention trials with patients with IBD between 77% and 86%, with lower rates in more recent trials, and no linear correlation between trial completion and duration. Lower completion rates were associated with the inclusion and frequency of fecal sampling, having a study duration of 4–8 wk, and having low RoB in the handling missing data. Higher completion was found in trials that did not include fecal sampling and did not use nonmonetary motivation strategies.

The results are highly heterogeneous, and further assessments of more homogeneous trials focusing on the most recent trials conducted by current research practices are required. Still, future dietary intervention trials may improve trial completion by reconsidering the feasibility of the intervention content and fecal sampling, or accounting for expected attrition rates during the design phase. Our findings suggest accounting for an additional 22% of participants in the sample size estimation and 33% if planning fecal sampling.

## Author contributions

The authors’ responsibilities were as follows – LG, BLH, þIH, RC: designed research; LG, CM, ZH, NFR: conducted research; LG, SRP: performed statistical analysis; LG: wrote paper; LG: had primary responsibility for final content; and all authors: read and approved the final manuscript.

## Data availability

The literature search, data described in the manuscript, code book, and analytic code will be made available on reasonable request to the first author.

## Declaration of generative AI and AI-assisted technologies in the writing process

No use of generative AI or AI-assisted technologies was used in the writing process.

## Funding

This project has received funding from University Hospital of Southern Denmark. The Parker Institute, Bispebjerg and Frederiksberg Hospital is supported by a core grant from the Oak Foundation (OFIL-24-074). Vibeke Andersen is supported by Sundhedsdonationer (2024-0379). The supporting sources had no involvement or restrictions regarding publication.

## Conflict of interest

VA has served as advisory board member for MSD (Merck). All other authors declare no conflict of interest.
