# Supplementary material for: Study Design Complexity and Participant Completion in Dietary Trials for Inflammatory Bowel Disease: A Systematic Review and Metaresearch Study
Source: Adv Nutr. 2026 Mar 11;17(4):100614. doi: 10.1016/j.advnut.2026.100614 (PMC13068548; doi:10.1016/j.advnut.2026.100614)
Supplement: Multimedia component 1 [file mmc1.pdf]

Supplementary material to:

Study Design Complexity and Participant Completion in Dietary Trials for Inflammatory Bowel Disease: A Systematic Review and Meta-Research Study

First Author: Laura Gregersen

Supplementary material legends

Supplementary material S1: Study protocol (pdf)

Supplementary material S2: PRISMA checklist

Supplementary material S3: Table 1 (flexible)

Supplementary material S4: Main outcome measures and findings

Supplementary material S5: Differential attrition sensitivity analysis

Supplementary material S6: Risk of bias summary

Supplementary material S7: Stratified sensitivity analyses – results from trials published in 2010-2024

Supplementary material S8: Stratified sensitivity analyses – results from trials published before 2010

Supplementary material to:

Study Design Complexity and Participant Completion in Dietary Trials for Inflammatory Bowel Disease: A Systematic Review and Meta-Research Study

First Author: Laura Gregersen

Supplementary material S1: Study protocol

Association between study design complexity on completion rates of participants with inflammatory bowel disease in dietary intervention trials: Protocol for a meta-research study

Laura Gregersen[1,2,3], Zainab Hikmat[1,2], Nathalie Fogh Rasmussen[1,2], Caroline Moos[4], Andreas Kristian Pedersen[4], Berit Heitmann[5], Vibeke Andersen[1,2], Þórhallur Ingi Halldórsson[6], Robin Christensen[3,7]

Author affiliations:
1 Molecular diagnostics and Clinical Research Unit, Department of Regional Health Research, University of Southern Denmark, Odense, Denmark.
2 Department for Blood samples, Biochemistry and Immunology, Hospital of Southern' Denmark, Aabenraa.
3 Section for Biostatistics and Evidence-Based Research, the Parker Institute, Bispebjerg and Frederiksberg Hospital, Copenhagen, Denmark.
4 Clinical Research Department, Hospital Sønderjylland, Aabenraa, Denmark.
5 Research Unit for Dietary Studies, the Parker Institute, Bispebjerg and Frederiksberg Hospital, Copenhagen, Denmark.
6 Faculty of Food and Science, University of Iceland, Reykjavik
7 Research Unit of Rheumatology, Department of Clinical Research, University of Southern Denmark, Odense University Hospital, Denmark.

Corresponding author: PhD fellow Laura Gregersen, M.Sc., e-mail: laura.gregersen@rsyd.dk

Registration
In accordance with the guidelines, our systematic review protocol will be registered with the International Prospective Register of Systematic Reviews (PROSPERO) and registration number will be included in the final manuscript.

Contributors
LG, BH, ÞIH, VA, and RC participated in the conception and design of this protocol, including draft search strategy development. LG, CM, and RC participated in search strategy development. LG will perform the search and selection in collaboration with ZH, NFR and CM. LG, ZH, NFR and CM will retrieve the data and participate in resolving potential agreements about eligibility. RC provided statistical advice for the design. All authors drafted and critically reviewed this manuscript and approved the final version.

Amendments
In the event of protocol amendments, we will give the date of each amendment, describe the change and give rationale in this section. Changes will not be incorporated into the original protocol.

Ethics and dissemination
As this review will be based on published data, a specific ethical clearance is not required. The results will be published in peer-reviewed journals.

Support
The project is supported by Hospital Sønderjylland, Region of Southern Denmark, and Knud og Edith Eriksens Mindefond. The Parker Institute, Bispebjerg and Frederiksberg Hospital is supported by a core grant from the Oak Foundation (OCAY-18-774-OFIL). Sponsors did not participate in developing the protocol.

Protocol version

Protocol version 1. This protocol has been approved by Robin Christensen, Vibeke Andersen, and Þórhallur Ingi Halldórsson on 30. June 2022.

Laura Gregersen Digitalt signeret af Laura Gregersen
Dato: 2022.06.30 13:29:21 +02'00'

Laura Gregersen, PhD fellow

INTRODUCTION

Background

Research on research (also known as meta-research) is a growing area of investigations aiming to improve scientific evidence and research credibility itself by evaluating reasons for systematic biases and identifying best practice in each step of the research process from study design to dissemination of results [1-3]. This helps to avoid research waste and ensure the best usage of resources. Methodological research seek to take a bird's-eye perspective on the methodology used in a specific research area to address systematic biases or scarcities in established methods rather than criticising individual studies [4].

Inflammatory Bowel Disease (IBD), covering the two main subtypes ulcerative colitis (UC) and Crohn's disease, is an autoimmune disease characterised by chronic intestinal inflammation which manifests as abdominal pain, diarrhoea and rectal bleeding. Due to the need of life-long treatment, IBD pose a heavy disease burden on the healthcare community [5] as well as the individual [6]. There is a growing demand for dietary recommendations to manage the disease course among patients and health care facilities [7, 8]. This has led to an increasing number of trials assessing the effect of diet or dietary supplements in IBD, but the evidence of potential beneficial effects of diet on IBD management is still scarce.

Controlled dietary trials are challenged by the risk of low completion rate and low adherence to the intervention diet as dietary behaviour is impossible to control according to standards for controlled clinical trials. Accounting for feasibility in the design and planning of dietary research is a key step in improving research standards. Low compliance to the protocol and low completion rates may result in inadequately powered trials and compromise study results [9, 10]. Feasibility and protocol adherence is limited by not only research resources but also by participants ability and willingness to participate and complete the study. Among factors not directly related to study design that may affect participant completion rate is diet accessibility, relevance of trial objectives, dietary restrictions, and frequency of study visits.

Rationale & Evidence-based research

With a growing body of contradictory results on the effects of diets on IBD symptoms and low completion rates from such studies, the importance of good internal validity (with a low risk of bias) and feasible trials has become apparent. To ensure high-quality trials and maximize the use of resources, it is important to meet the target sample size by ensuring protocol adherence and trial completion. However, to our knowledge, study design factors predicting adherence to dietary research projects in IBD have not been reviewed systematically. Hence, the aim of this meta-research project is to evaluate the completion rate in dietary intervention trials in patients with IBD, and to identify design factors that is associated with the trial completion rate.

However, to reduce research waste and further justify this current evidence synthesis study and to uncover most (if not all) existing systematic reviews [11] regarding reviews of factors

predicting drop-out from clinical trials, we performed a pragmatic search of existing and recent systematic reviews (search date: 5. February 2022), in accordance to the Evidence-Based Research principles [12, 13]. The search was carried out in PubMed, PROSPERO registry and Cochrane Library. The search strategy in PROSPERO and Cochrane Library included “drop-out” or “drop-outs” in the study title. The search in PubMed included the following “Boolean terms” filtered by reviews and systematic reviews:

Effect: drop-out*[ti] *OR* dropout*[ti] *OR* "drop out"[ti]

Design/Methodology: "Cochrane Database Syst Rev"[jour] *OR* meta-analysis[pt] *OR* “systematic review”[pt] *OR* meta-analys*[pt] *OR* meta-analys*[ti] *OR* metaanalys*[ti] *OR* meta-regress*[tiab] *OR* metaregress*[tiab] *OR* meta*[tiab] *OR* epidemiology*[tiab]

A second search was carried out in PubMed on xx. May 2022 using the following “Boolean terms”, filtered by reviews and systematic reviews, and limited to not including ‘animal*’, ‘mouse’, or ‘mice’ in the title:

Exposure: diet*[tiab] OR food*[tiab] OR eating*[tiab] OR meal*[tiab]

Effect: withdrawal*[ti] OR attrition[tiab]

Design/Methodology: "Cochrane Database Syst Rev"[jour] *OR* meta-analysis[pt] *OR* “systematic review”[pt] *OR* meta-analys*[pt] *OR* meta-analys*[ti] *OR* metaanalys*[ti] *OR* meta-regress*[tiab] *OR* metaregress*[tiab] *OR* meta*[tiab] *OR* epidemiology*[tiab]

After removal of duplicates, these searches resulted in 76 articles and 6 planned systematic reviews registered on PROSPERO (see full list of citations in appendix B). Of these, 11 articles were considered relevant judged by the title and abstract as they assessed reasons for drop-outs in intervention trials. Six articles assessed behavioural interventions (e.g. exercise) in other chronic diseases [14-17], cancer [18], or across various populations [19], and four assessed medication or pain management across various conditions [20-23]. Excluded search results included systematic reviews reporting drop-out rates but not analysing potential causes for drop-outs, systematic reviews assessing psychotherapy, behavioural therapy or treatment for mental or psychological disorders, and reviews assessing single-arm trials only, or aiming to statistically assess approaches to missing data. An overview of the potentially relevant systematic reviews is presented in **Table 1**.

To collect systematic reviews of potentially eligible trials for the present meta-epidemiological review, we further performed a scoping review of systematic reviews assessing dietary intervention trials in IBD management (search date: 14. February 2022) using a pragmatic search in PubMed [12]. This search resulted in 196 results after removal of duplicates of which 31 were considered relevant judged by the title and abstract (i.e., were systematic reviews assessing the effect of specified or unspecified diets or specified dietary components on IBD symptoms or disease activity). The pragmatic search strategy and list of the 31 relevant articles is in appendix C.

Table 1. Overview of related systematic reviews presented in the descending order of publication date

Jeon, S.R., D. Nam, and T.H. Kim, Dropouts in randomized clinical trials of Korean medicine interventions: a systematic review and meta-analysis. Trials, 2021. 22(1): p. 176.
Jabardo-Camprubí, G., et al., Drop-out ratio between moderate to high-intensity physical exercise treatment by patients with, or at risk of, type 2 diabetes mellitus: A systematic review and meta-analysis. Physiol Behav, 2020. 215: p. 112786.
Dennett, R., et al., Adherence and drop-out in randomized controlled trials of exercise interventions in people with multiple sclerosis: A systematic review and meta-analyses. Mult Scler Relat Disord, 2020. 43: p. 102169.
Meyerowitz-Katz, G., et al., Rates of Attrition and Dropout in App-Based Interventions for Chronic Disease: Systematic Review and Meta-Analysis. J Med Internet Res, 2020. 22(9): p. e20283.
Vancampfort, D., et al., Dropout from physical activity interventions in people living with HIV: a systematic review and meta-analysis. AIDS Care, 2017. 29(5): p. 636-643.
Cramer, H., et al., A Systematic Review and Meta-Analysis Estimating the Expected Dropout Rates in Randomized Controlled Trials on Yoga Interventions. Evid Based Complement Alternat Med, 2016. 2016: p. 5859729.
Corona, G., et al., First-generation phosphodiesterase type 5 inhibitors dropout: a comprehensive review and meta-analysis. Andrology, 2016. 4(6): p. 1002-1009.
Zhong, X., et al., Effects of three injectable antidiabetic agents on glycaemic control, weight change and drop-out in type 2 diabetes suboptimally controlled with metformin and/or a sulfonylurea: A network meta-analysis. Diabetes Res Clin Pract, 2015. 109(3): p. 451-60.
Steins Bisschop, C.N., et al., Control group design, contamination and drop-out in exercise oncology trials: a systematic review. PLoS One, 2015. 10(3): p. e0120996.
Makatsori, M., et al., Dropouts in sublingual allergen immunotherapy trials - a systematic review. Allergy, 2014. 69(5): p. 571-80.

Research question

How large is the proportion of patients with inflammatory bowel disease who complete the trial period when enrolled in randomised dietary intervention trials (independent of group/arm), and which study design factors can potentially explain trial completion in patients with these conditions?

Aim

The aim of this meta-research study is to analyse the association between trial complexity and the completion rates in dietary intervention trials among participants with IBD.

METHODS

As guidelines for reporting of methodological research has not yet been developed [3], this review protocol has been prepared according to the 2015 Preferred Reporting Items for Systematic Reviews and Meta-Analyses Protocols guidelines (PRISMA-P) [24]. The PRISMA-P checklist is provided in appendix D.

Eligibility criteria

Studies will be scrutinised for eligibility based on the following criteria outlined below:

Eligible conditions: Inflammatory bowel disease (IBD), i.e., any diagnose of Crohn's disease, ulcerative colitis, ulcerative pancolitis, or proctitis/proctocolitis.

Eligible populations: Studies assessing patients above the age of four of all cultural backgrounds with one or more of the eligible conditions will be included. Interventions assessing babies and toddlers aged 0-4 years will not be considered eligible.

Eligible interventions: Of interest are studies examining any dietary regimen and/or orally distributed foods, drinks, or dietary supplements. Studies examining the effects of parenteral nutrition, exclusive enteral nutrition, encapsulated supplements to habitual diet, or diets fully controlled by hospitalization (i.e., the hospital provides all meals) will be excluded.

Eligible comparators: Studies comparing the exposure to habitual diet, placebo or sham diet, standard care or placebo supplement will be included. Data from control groups without the eligible conditions will not be extracted for the present review.

Eligible study designs: We will include all individual groups (arms) from randomised clinical trials (RCTs) exploring the effects of a dietary intervention (or control/unexposed group). Pilot and feasibility studies of relevant RCTs will be included. Inclusion criteria will be restricted to English, Spanish and Scandinavian languages, but a list of possible relevant titles reported in other ineligible languages will be provided in supplementary files. No restrictions by type of setting or publication date will be applied.

Information sources

We will search MEDLINE (OVID interface, 1948 onwards), EMBASE (OVID interface, 1980 onwards), and CINAHL Healthcare Databases Advanced Search (Cumulative Index to Nursing and Allied Health Literature; 1981 to current). Additionally, we will browse reference lists of related systematic reviews (appendix B), included studies, and relevant reviews identified through the search. We will supplement our electronic database search by searching for trial protocols through metaRegister (http://www.controlled-trials.com/ mrct/), International Clinical Trials Registry Platform Search Portal, and ClinicalTrials.gov for ongoing or recently completed trials [25]. Finally, to search grey literature, we will circulate our bibliography of relevant studies to experts identified

by the review authors, and browse the databases Open Grey (http://opengrey.eu/) and Grey literature Report (https://www.greylit.org/), Google Scholar (first 100 results when sorted by relevance), ESPEN guidelines, patient unions and similar online resources. Additionally, we will apply backward and forward chaining on articles from our bibliography published less than five years prior to completed full text screen. Search words for all databases will be described in the publication.

Search strategy

We will develop our literature search strategies using database specific subject headings and corresponding keywords related to included study criteria within the three blocks: IBD diagnose, RCT study design, and diet. For the study design search block, the validated Cochrane search strategy to search for human study RCT's in MEDLINE, EMBASE, and Cinahl will be used [26]. The IBD diagnose search block will be developed based on the IBD search from a recent Cochrane review assessing surgical therapies in IBD [27], and the diet search block will be inspired by a Cochrane review assessing dietary interventions in IBD [28]. The search strategy will be developed with input from all project team members, and then have it peer reviewed by an information specialist not otherwise associated with the project using the PRESS standard [29]. Our MEDLINE search strategy is summarised in appendix E. The MEDLINE search strategy will be adapted to the subject headings and syntax of the other databases.

Data management

Citation, abstracts and full text articles from all search results will be uploaded to Covidence software to facilitate collaboration between reviewers during the study selection process and creation of selection flow diagram. Duplicates will be removed using automated de-duplication in Covidence software, recently rated among the most accurate de-duplication methods for conducting a systematic review [30], prior to the selection process.

Study selection process

Four reviewers (ZH, NFR, CM, LG) will screen and select studies with every article being screened by at least LG and one other reviewer. We will develop screening questions for first and second assessment based on inclusion and exclusion criteria. To pilot test, customise, and refine the screening questions, the reviewers will undertake a calibration exercise with eight randomly selected search results prior to the formal screening process. The reviewers will independently screen all titles and abstracts yielded by the search against the screening questions, and then screen full reports for all titles that appear to meet the inclusion criteria or where there is any uncertainty and decide whether these meet the inclusion criteria. Disagreements will be solved through discussions with a third review author. We will seek additional information from study authors when necessary to resolve questions about eligibility. Neither of the review authors will be blinded to the

journal titles or to the study authors or institutions. The selection process will be summarised in a flow diagram, and reasons for study exclusion will be described.

Data collection process

Two reviewers will extract data, and all relevant sources will be used. Where available, the journal article will be used as the primary source, as this has been published after appropriate peer review. We will develop a data extraction sheet using Covidence software for data extraction prior to the data collection process. To pilot and refine the data extraction sheet, the reviewers will undertake a calibration exercise using eight randomly selected titles from our search. Based on the final data extraction sheet, the reviewers will independently extract data from all selected studies. Disagreements will be resolved through discussion and in case of uncertainties, additional information will be sought from study authors.

Data items

Based on the data extraction sheet, we will collect data on:

- *Study identification:* Year of publication, name of first author, country of origin, recruitment period, trial registration number.
- *Study and participant characteristics:* Study duration, intervention diet(s), comparator diet(s), primary outcome measure, the most clinically relevant dichotomous outcome, between-group effect size, sex, age, IBD diagnose, dietary compliance measure(s).
- *Sample size, by study arm:* No. of participants invited, randomised, and completed each study arm, no. participants completed each follow-up time point.
- *Additional participant flow, by study arm:* Estimated enrolment, registered adverse events, and reported reasons for dropouts according to the publications or clinical trials registration (will be reported in supplementary materials only).
- *Moderator variables for study design, by study arm:* Intervention diet and/or supplementation type, comparator diet/supplement, study design, study duration, the inclusion of biologic samples (stool, blood and urine), the delivery method of intervention foods, dietary instructions included, motivational actions included.
- *Moderator variables, baseline participant characteristics, by study arm:* Sex, age, diagnose, study arm, disease duration, disease activity, the use of biologic treatment, blinding strategies.

Data will be collected by study arm. If data is not reported by study arm, they will be collected and reported by study within each study arm, i.e. the present data will be identical in all study arms from the same study.

Outcome domains and measurements

The primary outcome will be the completion rate of participants included in the Intention-To-Treat population, extracted as reported by study arms. Completion will be defined as participants with complete data at the last follow-up according to the flowchart, abstract, or text fields in the primary publication. If this is not reported, completion will be defined as those included in the primary analysis. If sufficient information is not provided, data will be sought from the clinical trial registration. Trials will not be included in the meta-analysis, if data on participant flow is not available. Published reasons for dropping out of the trials will be collected and subsequently reported in supplementary materials.

Risk of bias

To evaluate the validity of included studies, we will incorporate a critical appraisal of each study. Each reviewer will make judgement independently, and disagreements will be resolved by discussion. Randomised controlled trials will be evaluated using the Cochrane Collaboration tool for assessing risk of bias [31]. This covers sequence generation, allocation concealment, blinding, incomplete outcome data, and selective reporting. Each section will be rated 'high risk', 'low risk' or 'unclear' if data is insufficient according to the criteria [31]. If at least one of the sections are rated 'high risk', the study will be considered at high risk of bias. If all sections are rated 'low', the trial will be considered at low risk of bias.

Reporting biases: In order to evaluate possible reporting bias, we will determine whether the protocol of the RCT was published before recruitment of patients was started. For studies published after July 1st, 2005, we will screen the Clinical Trial Register at the International Clinical Trials Registry Platform of the World Health Organisation (http://apps.who.int/trialssearch). The potential for reporting bias will be further explored by funnel plots if ≥10 studies are available.

Moderator variables (sub-group and meta-regression analyses)

Meta-regression analyses will be performed across all included study arms and within the study design complexity variables and baseline patient characteristics with corresponding categories outlined in table 3 and 4. Study design complexity variables are selected based on factors that may affect efficacy in nutrition education programmes identified through review of relevant publications (intervention content, study design, study duration, and motivation/instructions from dietician) [32, 33], and based on discussion with patient representatives (intervention content incl. blinding, study duration, biologic sample collection, motivation, and reward). Baseline patient characteristics are chosen based on common descriptive information about study arm, disease course and severity identified through relevant articles to assess potential associations between completion rate and disease course at baseline.

Dietary regimen will be reported as restrictive or solely additive. *Restrictive diets* will be those changing habitual diet by restricting specific dietary components or food items. Restrictive

diets including the supplementation of specific food items will also be defined as restrictive diets. *Additive diets* will be those trials adding dietary supplements and/or food items to habitual diet.

Diet provided will be reported as trials providing or not providing all food items or supplements relevant for the intervention. *Relevant food items* will be defined as foods or supplements included in the intervention trial other than habitual diet. If participants are required to purchase additional ingredients to comply with the intervention, the present study arm will be defined as 'some' or 'no' food items provided.

Blinding of patients will be reported as adequate or inadequate. Blinding will be defined as *adequate* if participants were kept unaware of their treatment and the blinding procedure was described in the study material available for review. If this was not the case, the blinding will be defined as *inadequate*.

Table 3. Study design complexity variables and categories for the meta-analyses

Variables	Categories	Expectations
Dietary regimen*	Restrictive<br>Additive	Higher completion rate with additive diet
Study design	Cross-over design<br>Other randomised controlled trial designs	Higher completion rate with 'other' design.
Total study duration	< 4 weeks (1 month)<br>≥ 4 weeks (1 month)	Higher completion rate with shorter duration. Will be further investigated as a continuous variable.
Faecal samples included	Yes<br>No	Higher completion rate with no biologic samples collection
Blood samples included	Yes<br>No	
Urine samples included	Yes<br>No	
Diet provided*	All relevant food items provided<br>Some or no food items provided/not specified	Higher completion rate with diet provided
Diet instructions	By dietician<br>By other or unclear/not specified	Higher completion rate with instructions from dietician
Motivational actions included	Yes<br>No/not specified	Higher completion rate with motivation during the trial
Reward for participation included	Yes<br>No/unclear	Higher completion rate with reward for completion

* 'dietary regimen', and 'diet provided' are elaborated in section 'Moderator variables'.

Table 4. Baseline characteristics variables for the meta-analyses

Variables	Variable type
Females	Proportion
Age	Continuous
Intervention group	Proportion
UC diagnose	Proportion
Disease duration	Continuous
Disease activity	Continuous
Biologic treatment usage	Proportion
Adequate blinding of patients*	Proportion

* blinding of patients is elaborated in section 'Moderator variables' above.

Data synthesis

Study characteristics will be presented descriptively to give an overview of the included studies.

Summary measures: To synthesise the completion rate estimates from the different arms, each proportion will be transformed using the logit function as proportions is a realisation of binomially distributed random variables.

Evidence synthesis: The overall completion rate and its influencing variables will be estimated using a mixed effect model. This is possible following the assumption by Dersimonian and Laird in their famous 1986 paper that the effect size for each study follows the following formula:

$$\boldsymbol{\theta_i = \theta + e_i},$$

where $e_i \sim N(0, \tau + \sigma_i)$. This formula states that these effect sizes is identical to a classic random effect model from variance analysis, hence the analysis can be extended to a mixed effect model as follows:

$$\boldsymbol{\theta_i = \theta + X_i\beta + Y_i\alpha + e_i},$$

where β and α are the matrices of the fixed and random effects, respectively. Thereby the estimate of the overall completion rate will be more precise as the influencing exposures can and will be estimated and adjusted for.

In order to assess the heterogeneity, the variance components will be estimated from the mixed effect model and by using forest plots (and potentially funnel plots too [34]). This is possible as the Kendall's τ^2 is equal to the variance components which can be estimated using restricted maximum likelihood (REML) estimation ($\sigma_i = 1$). The method provides confidence intervals (CIs) for τ^2 with the coverage being the nominal level and weighs the studies using standard errors (SEs). The I^2-statistics for the τ^2 estimation will be calculated in a similar manner using the delta method. The cut-off value for high heterogeneity will be set to 75% for the I^2

statistic, but the other methods will also be taken into account. Margins for the categorical variables will be further estimated from the mixed effect model [35].

All estimates will be reported after an appropriate conversion with their corresponding 95% CI.

A two-sided 95% Confidence Interval not overlapping "the null" and a p-value < 0.05 will be considered as possibly statistically significant. Bayesian approaches may apply for further sensitivity analyses. All analyses will be conducted in the statistical software STATA or R statistics.

Adjusting for meta-confounding: Inference coming from meta-epidemiological research is observational (and not randomized); meta-epidemiological studies are useful for informing the design of clinical trials only if they provide evidence that a particular feature of trial design causes bias. We will attempt to apply "de-confounding approaches" that can potentially prevent the risk of bias from residual confounding in observational studies [36]. Estimates quantifying an association will be reported along with its corresponding E-value to assess the robustness of any proposed causal association to confounding.

Confidence in cumulative estimate

The quality of evidence for the outcome will be assessed across the domains of risk of bias, consistency, directness, precision and publication bias based on the Grading of Recommendations Assessment, Development and Evaluation (GRADE) working group methodology. Quality will be assessed as 'high' (further research is unlikely to change our confidence in the outcomes), 'moderate' (further research is likely to have an important impact on our confidence in the outcomes), 'low' (further research is very likely to have an important impact on our confidence in the outcomes), or 'very low' (very uncertain about the estimate of outcomes).

Appendices

A: Illustration of tables and figures for publication of study results incl. supplementary tables.

B: Full list of citations from pragmatic search of existing studies.

C: List of citations of systematic reviews of potentially eligible trials and list of relevant trials incl. the search strategy.

D: PRISMA checklist.

E: MEDLINE search strategy.

Appendix A. Illustration of tables and figures for publication of study results

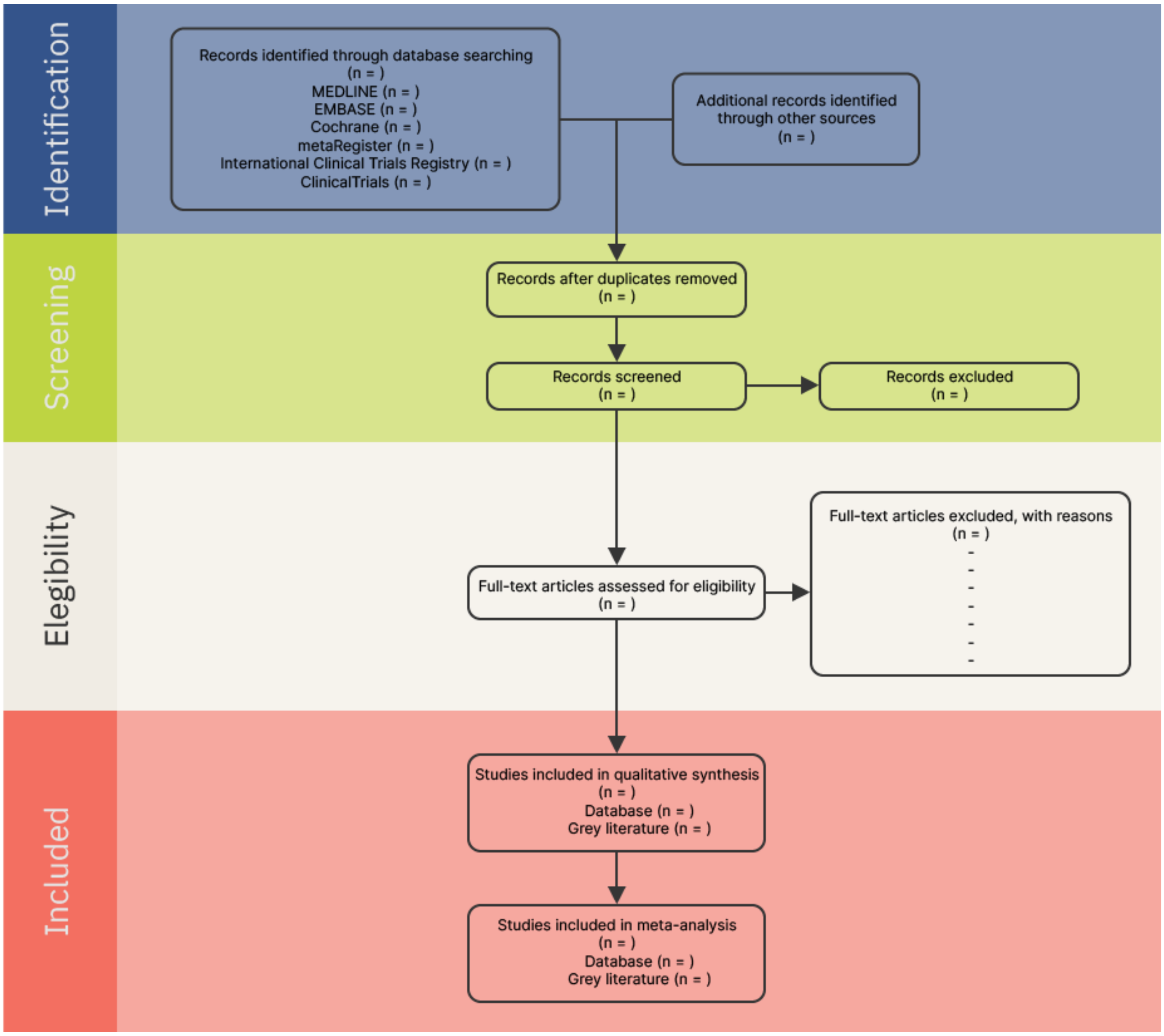

Figure 1. Flow chart of trial inclusion.

Table 1. Study characteristics presented by specific trial and individual arms (groups)

Study	Study origin	Study design	Recruitment period	Trial duration	Primary outcome (dichot.) ‡	Study arm	Diet content and type*	Population (% females)	Age
Last name yyyy	Country	Cross-over/ parallel/ factorial	mm yyyy – mm yyyy	xx d/w/ mo.	[domain] [00.00]/effect size (ES) ([domain], [00.00], ES)	1	[Placebo type]	n [IBD-type] or healthy (xx %)	[xx-xx]
						2	[diet] Additive/ Restrictive	n [IBD-type] (xx %)	[xx-xx]
Last name yyyy	Country	Cross-over/ parallel/ factorial	mm yyyy – mm yyyy	xx d/w/ mo.	[domain] [00.00]/effect size (ES) ([domain], [00.00], ES)	1	[Placebo type]	n [IBD-type] or healthy (xx %)	[xx-xx]
						2	[diet] Additive/ Restrictive	n [IBD-type] (xx %)	[xx-xx]
Last name yyyy	Country	Cross-over/ parallel/ factorial	mm yyyy – mm yyyy	xx d/w/ mo.	[domain] [00.00]/effect size (ES) ([domain], [00.00], ES)	1	[Placebo type]	n [IBD-type] or healthy (xx %)	[xx-xx]
						2	[diet] Additive/ Restrictive	n [IBD-type] (xx %)	[xx-xx]
Last name yyyy	Country	Cross-over/ parallel/ factorial	mm yyyy – mm yyyy	xx d/w/ mo.	[domain] [00.00]/effect size (ES) ([domain], [00.00], ES)	1	[Placebo type]	n [IBD-type] or healthy (xx %)	[xx-xx]
						2	[diet] Additive/ Restrictive	n [IBD-type] (xx %)	[xx-xx]
Last name yyyy	Country	Cross-over/ parallel/ factorial	mm yyyy – mm yyyy	xx d/w/ mo.	[domain] [00.00]/effect size (ES) ([domain], [00.00], ES)	1	[Placebo type]	n [IBD-type] or healthy (xx %)	[xx-xx]
						2	[diet] Additive/ Restrictive	n [IBD-type] (xx %)	[xx-xx]
Last name yyyy	Country	Cross-over/ parallel/ factorial	mm yyyy – mm yyyy	xx d/w/ mo.	[domain] [00.00]/effect size (ES) ([domain], [00.00], ES)	1	[Placebo type]	n [IBD-type] or healthy (xx %)	[xx-xx]
						2	[diet] Additive/ Restrictive	n [IBD-type] (xx %)	[xx-xx]
Last name yyyy	Country	Cross-over/ parallel/ factorial	mm yyyy – mm yyyy	xx d/w/ mo.	[domain] [00.00]/effect size (ES) ([domain], [00.00], ES)	1	[Placebo type]	n [IBD-type] or healthy (xx %)	[xx-xx]
						2	[diet] Additive/ Restrictive	n [IBD-type] (xx %)	[xx-xx]
Last name yyyy	Country	Cross-over/ parallel/ factorial	mm yyyy – mm yyyy	xx d/w/ mo.	[domain] [00.00]/effect size (ES) ([domain], [00.00], ES)	1	[Placebo type]	n [IBD-type] or healthy (xx %)	[xx-xx]
						2	[diet] Additive/ Restrictive	n [IBD-type] (xx %)	[xx-xx]

‡domain, measure and the between-group effect size as published. If the primary outcome is not dichotomous, the most clinical important dichotomous outcome will be reported along with the primary outcome to interpret the influence from completion rate on the dichotomous outcome. *Diet type: restricted or additive. UC = Ulcerative Colitis, CD = Crohn's Disease, IBD = Inflammatory Bowel Disease.

Figure 2. Forrest plot of completion rate by study arm.

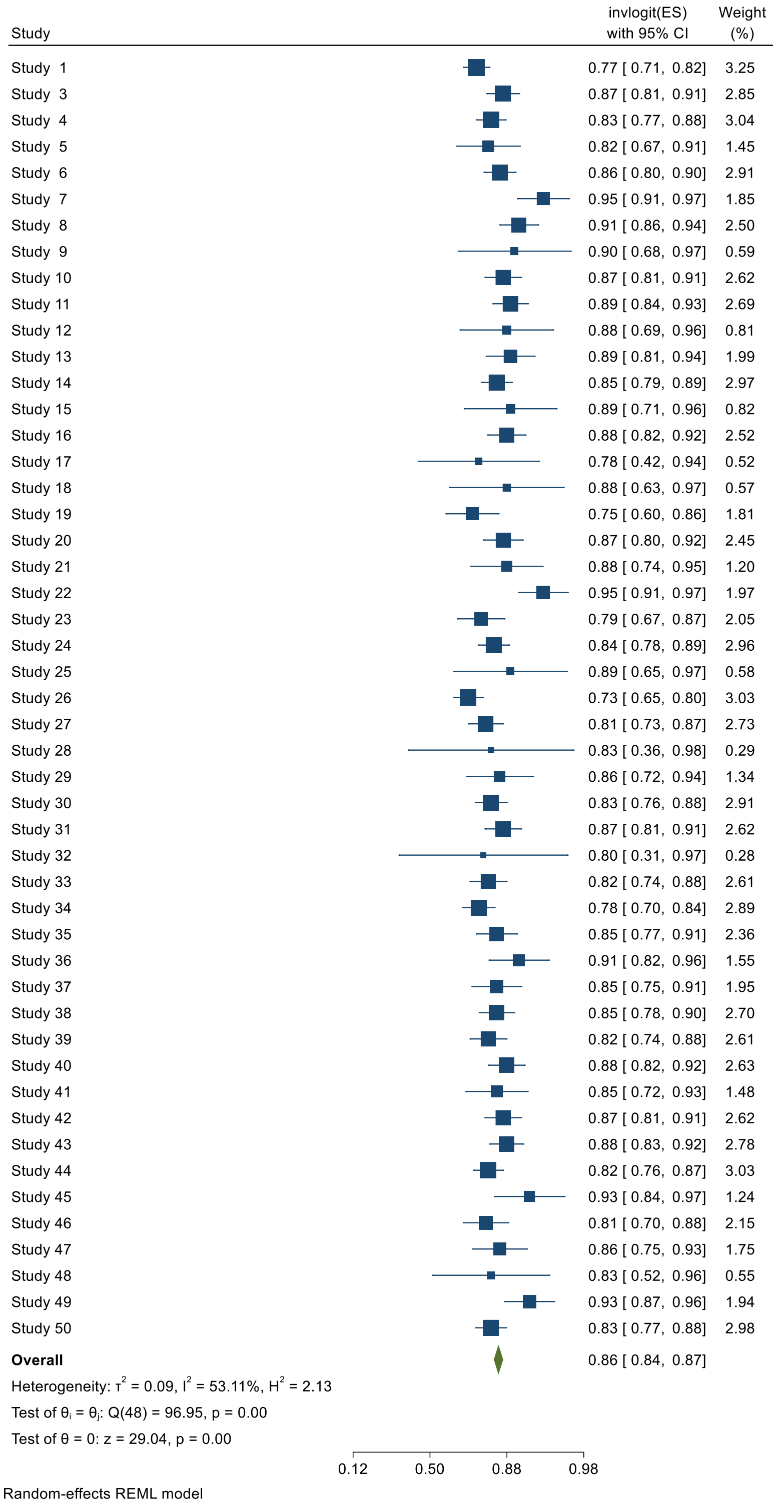

Study = study arm; invlogit = completion rate probability

Table 2. Results of the stratified meta-analysis for design characteristics modifying completion rate (univariate analysis). Fixed effects for trial arms while a random factor for the specific trial.

Analysis	Number of arms (trials)	Meta-analysis							E value
		Completion rate	95% CI		I²	τ^2	Relative difference	*P*-value for association	
Overall	00	00.0	00.0	00.0	00.0	00.0			00.0
Dietary regimen*					00.0	00.0	00 %	0.000	00.0
Restrictive	00	00.0	00.0	00.0					
Additive	00	00.0	00.0	00.0					
Study design					00.0	00.0	00 %	0.000	00.0
Cross-over	00	00.0	00.0	00.0					
Parallel/factorial	00	00.0	00.0	00.0					
Total study duration					00.0	00.0	00 %	0.000	00.0
< 4 weeks	00	00.0	00.0	00.0					
≥ 4 weeks	00	00.0	00.0	00.0					
Faecal sample incl.					00.0	00.0	00 %	0.000	00.0
Yes	00	00.0	00.0	00.0					
No	00	00.0	00.0	00.0					
Blood sample incl.					00.0	00.0	00 %	0.000	00.0
Yes	00	00.0	00.0	00.0					
No	00	00.0	00.0	00.0					
Urine sample incl.					00.0	00.0	00 %	0.000	00.0
Yes	00	00.0	00.0	00.0					
No	00	00.0	00.0	00.0					
Diet provided					00.0	00.0	00 %	0.000	00.0
All	00	00.0	00.0	00.0					
None/some	00	00.0	00.0	00.0					
Diet instructions					00.0	00.0	00 %	0.000	00.0
By dietician	00	00.0	00.0	00.0					
By others/unclear	00	00.0	00.0	00.0					
Motivation included					00.0	00.0	00 %	0.000	00.0
Yes	00	00.0	00.0	00.0					
No/unclear	00	00.0	00.0	00.0					

Figure 3. Visualisation of the meta-regression of completion rate on study duration.

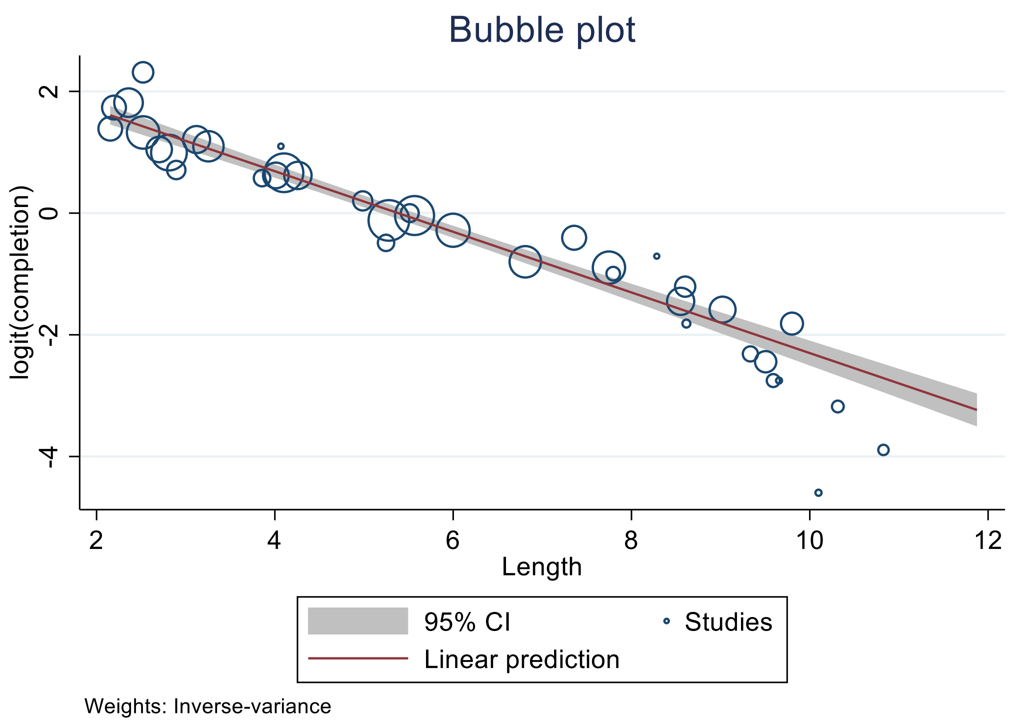

Table 3. Results of the meta-regression for participant baseline characteristics modifying completion rate (univariate analysis). Fixed effects for variables while random effects for the specific trial.

	Meta-analysis						
**Analysis**	**Completion probability (RR)**	**95% CI**		**I²**	**τ²**	***P*-value for association**	**E value**
Female*	00.0	00.0	00.0	00.0	00.0	0.000	00.0
Age	00.0	00.0	00.0	00.0	00.0	0.000	00.0
UC diagnose*	00.0	00.0	00.0	00.0	00.0	0.000	00.0
Disease duration	00.0	00.0	00.0	00.0	00.0	0.000	00.0
Disease activity	00.0	00.0	00.0	00.0	00.0	0.000	00.0
Biologic treatment*	00.0	00.0	00.0	00.0	00.0	0.000	00.0
Intervention group*	00.0	00.0	00.0	00.0	00.0	0.000	00.0
Adequate blinding of patients‡*	00.0	00.0	00.0	00.0	00.0	0.000	00.0

*Binary variables will be analysed with as the proportion of the presented reference group. ‡Adequate blinding will be assumed in studies describing that they keep the participants unaware of the treatment content.

Supplementary table S1. Trial withdrawals and reported reasons for withdrawal.

Study	Estimated enrolment (n)	No. randomised (n)	No. completed (n)	Reported reasons for withdrawal	Adverse events
Last name yyyy	[no.]	[no.]	[no.]	[description: (n)]<br>[description: (n)]<br>…<br>Adverse events: [n]	[description]
Last name yyyy	[no.]	[no.]	[no.]	[description: (n)]<br>[description: (n)]<br>…<br>Adverse events: [n]	[description]
Last name yyyy	[no.]	[no.]	[no.]	[description: (n)]<br>[description: (n)]<br>…<br>Adverse events: [n]	[description]
Last name yyyy	[no.]	[no.]	[no.]	[description: (n)]<br>[description: (n)]<br>…<br>Adverse events: [n]	[description]
Last name yyyy	[no.]	[no.]	[no.]	[description: (n)]<br>[description: (n)]<br>…<br>Adverse events: [n]	[description]
Last name yyyy	[no.]	[no.]	[no.]	[description: (n)]<br>[description: (n)]<br>…<br>Adverse events: [n]	[description]
Last name yyyy	[no.]	[no.]	[no.]	[description: (n)]<br>[description: (n)]<br>…<br>Adverse events: [n]	[description]
Last name yyyy	[no.]	[no.]	[no.]	[description: (n)]<br>[description: (n)]<br>…<br>Adverse events: [n]	[description]
Last name yyyy	[no.]	[no.]	[no.]	[description: (n)]<br>[description: (n)]<br>…<br>Adverse events: [n]	[description]

Supplementary table S2. Results of the stratified meta-analysis for design characteristics modifying completion rate (univariate analysis). Fixed effects for trial arms while a random factor for the specific trial.

Analysis	Number of arms (trials)	Meta-analysis<br>Completion rate	95% CI		I^2	τ^2	Relative difference	*P*-value for association	E value
Overall	00	00.0	00.0	00.0	00.0	00.0			00.0
Dietary regimen*					00.0	00.0	00 %	0.000	00.0
Restrictive	00	00.0	00.0	00.0					
Additive	00	00.0	00.0	00.0					
Study design					00.0	00.0	00 %	0.000	00.0
Cross-over	00	00.0	00.0	00.0					
Parallel/factorial	00	00.0	00.0	00.0					
Total study duration					00.0	00.0	00 %	0.000	00.0
< 4 weeks	00	00.0	00.0	00.0					
≥ 4 weeks	00	00.0	00.0	00.0					
Faecal sample incl.					00.0	00.0	00 %	0.000	00.0
Yes	00	00.0	00.0	00.0					
No	00	00.0	00.0	00.0					
Blood sample incl.					00.0	00.0	00 %	0.000	00.0
Yes	00	00.0	00.0	00.0					
No	00	00.0	00.0	00.0					
Urine sample incl.					00.0	00.0	00 %	0.000	00.0
Yes	00	00.0	00.0	00.0					
No	00	00.0	00.0	00.0					
Diet provided					00.0	00.0	00 %	0.000	00.0
All	00	00.0	00.0	00.0					
None/some	00	00.0	00.0	00.0					
Diet instructions					00.0	00.0	00 %	0.000	00.0
By dietician	00	00.0	00.0	00.0					
By others/unclear	00	00.0	00.0	00.0					
Motivation included					00.0	00.0	00 %	0.000	00.0
Yes	00	00.0	00.0	00.0					
No/unclear	00	00.0	00.0	00.0					
Urine sample incl.					00.0	00.0	00 %	0.000	00.0
Yes	00	00.0	00.0	00.0					
No/unclear	00	00.0	00.0	00.0					

* Restrictive diets are those restricting specific dietary components or food items with or without adding dietary supplements or food items, and additive diets are those adding any supplement or food item without otherwise restricting the diet.

Supplementary table 3S. Results of the meta-regression for participant baseline characteristics modifying completion rate (multivariate analysis). Fixed effects for variables while random effects for the specific trial.

						Meta-analysis	
**Analysis**	**Completion probability (RR)**	**95% CI**		**I²**	**τ²**	***P*-value for association**	**E value**
Female*	00.0	00.0	00.0	00.0	00.0	0.000	00.0
Age	00.0	00.0	00.0	00.0	00.0	0.000	00.0
UC diagnose*	00.0	00.0	00.0	00.0	00.0	0.000	00.0
Disease duration	00.0	00.0	00.0	00.0	00.0	0.000	00.0
Disease activity	00.0	00.0	00.0	00.0	00.0	0.000	00.0
Biologic treatment*	00.0	00.0	00.0	00.0	00.0	0.000	00.0
Intervention group*	00.0	00.0	00.0	00.0	00.0	0.000	00.0
Adequate blinding of patients‡*	00.0	00.0	00.0	00.0	00.0	0.000	00.0

*Binary variables will be analysed with as the proportion of the presented reference group. ‡Adequate blinding will be assumed in studies describing that they keep the participants unaware of the treatment content.

Appendix B. Full list of citations from pragmatic search of existing studies.

Table 1. Full list of citations in alphabetic order by first author

1. Bakker EJM, Kox J, Boot CRL, Francke AL, van der Beek AJ, Roelofs P. Improving mental health of student and novice nurses to prevent dropout: A systematic review. J Adv Nurs. 2020;76(10):2494-509.
2. Barretto Dos Santos Lopes Batista K, Thiruvenkatachari B, O'Brien K. Intention-to-treat analysis: Are we managing dropouts and missing data properly in research on orthodontic treatment? A systematic review. Am J Orthod Dentofacial Orthop. 2019;155(1):19-27.e3.
3. Berlim MT, van den Eynde F, Tovar-Perdomo S, Daskalakis ZJ. Response, remission and drop-out rates following high-frequency repetitive transcranial magnetic stimulation (rTMS) for treating major depression: a systematic review and meta-analysis of randomized, double-blind and sham-controlled trials. Psychol Med. 2014;44(2):225-39.
4. Bortolotti F, Corazza G, Bartolucci ML, Incerti Parenti S, Paganelli C, Alessandri-Bonetti G. Dropout and adherence of obstructive sleep apnoea patients to mandibular advancement device therapy: A systematic review of randomised controlled trials with meta-analysis and meta-regression. J Oral Rehabil. 2021.
5. Brorson HH, Ajo Arnevik E, Rand-Hendriksen K, Duckert F. Drop-out from addiction treatment: a systematic review of risk factors. Clin Psychol Rev. 2013;33(8):1010-24.
6. Chatfield MD, Brayne CE, Matthews FE. A systematic literature review of attrition between waves in longitudinal studies in the elderly shows a consistent pattern of dropout between differing studies. J Clin Epidemiol. 2005;58(1):13-9.
7. Cooper AA, Conklin LR. Dropout from individual psychotherapy for major depression: A meta-analysis of randomized clinical trials. Clin Psychol Rev. 2015;40:57-65.
8. Corona G, Rastrelli G, Burri A, Serra E, Gianfrilli D, Mannucci E, et al. First-generation phosphodiesterase type 5 inhibitors dropout: a comprehensive review and meta-analysis. Andrology. 2016;4(6):1002-9.
9. Cramer H, Haller H, Dobos G, Lauche R. A Systematic Review and Meta-Analysis Estimating the Expected Dropout Rates in Randomized Controlled Trials on Yoga Interventions. Evid Based Complement Alternat Med. 2016;2016:5859729.
10. de Haan AM, Boon AE, de Jong JT, Hoeve M, Vermeiren RR. A meta-analytic review on treatment dropout in child and adolescent outpatient mental health care. Clin Psychol Rev. 2013;33(5):698-711.
11. de Haan AM, Boon AE, de Jong J, Vermeiren R. A review of mental health treatment dropout by ethnic minority youth. Transcult Psychiatry. 2018;55(1):3-30.
12. de Jong K, Conijn JM, Gallagher RAV, Reshetnikova AS, Heij M, Lutz MC. Using progress feedback to improve outcomes and reduce drop-out, treatment duration, and deterioration: A multilevel meta-analysis. Clin Psychol Rev. 2021;85:102002.
13. Dejong H, Broadbent H, Schmidt U. A systematic review of dropout from treatment in outpatients with anorexia nervosa. Int J Eat Disord. 2012;45(5):635-47.
14. Dennett R, Madsen LT, Connolly L, Hosking J, Dalgas U, Freeman J. Adherence and drop-out in randomized controlled trials of exercise interventions in people with multiple sclerosis: A systematic review and meta-analyses. Mult Scler Relat Disord. 2020;43:102169.
15. Dixon LJ, Linardon J. A systematic review and meta-analysis of dropout rates from dialectical behaviour therapy in randomized controlled trials. Cogn Behav Ther. 2020;49(3):181-96.
16. Dunn TL, Casey LM, Sheffield J, Newcombe P, Chang AB. Dropout from computer-based interventions for children and adolescents with chronic health conditions. J Health Psychol. 2012;17(3):429-42.
17. Edwards-Stewart A, Smolenski DJ, Bush NE, Cyr BA, Beech EH, Skopp NA, et al. Posttraumatic Stress Disorder Treatment Dropout Among Military and Veteran Populations: A Systematic Review and Meta-Analysis. J Trauma Stress. 2021;34(4):808-18.
18. Eskildsen A, Hougaard E, Rosenberg NK. Pre-treatment patient variables as predictors of drop-out and treatment outcome in cognitive behavioural therapy for social phobia: A systematic review. Nord J Psychiatry. 2010;64(2):94-105.
19. Firth N, Barkham M, Delgadillo J, Allery K, Woodward J, O'Cathain A. Socioeconomic Deprivation and Dropout from Contemporary Psychological Intervention for Common Mental Disorders: A Systematic Review. Adm Policy Ment Health. 2021.
20. Gersh E, Hallford DJ, Rice SM, Kazantzis N, Gersh H, Gersh B, et al. Systematic review and meta-analysis of dropout rates in individual psychotherapy for generalized anxiety disorder. J Anxiety Disord. 2017;52:25-33.
21. Ghorbani M, Hosseini FS, Yunesian M, Keramat A. Dropout of infertility treatments and related factors among infertile couples. Reprod Health. 2020;17(1):192.
22. Goetter EM, Bui E, Ojserkis RA, Zakarian RJ, Brendel RW, Simon NM. A Systematic Review of Dropout From Psychotherapy for Posttraumatic Stress Disorder Among Iraq and Afghanistan Combat Veterans. J Trauma Stress. 2015;28(5):401-9.
23. Gomeni R, Lavergne A, Merlo-Pich E. Modelling placebo response in depression trials using a longitudinal model with informative dropout. Eur J Pharm Sci. 2009;36(1):4-10.
24. Gregertsen EC, Mandy W, Kanakam N, Armstrong S, Serpell L. Pre-treatment patient characteristics as predictors of drop-out and treatment outcome in individual and family therapy for adolescents and adults with anorexia nervosa: A systematic review and meta-analysis. Psychiatry Res. 2019;271:484-501.

25. Gubbels J, van der Put CE, Assink M. Risk Factors for School Absenteeism and Dropout: A Meta-Analytic Review. J Youth Adolesc. 2019;48(9):1637-67.

26. Jabardo-Camprubí G, Donat-Roca R, Sitjà-Rabert M, Milà-Villarroel R, Bort-Roig J. Drop-out ratio between moderate to high-intensity physical exercise treatment by patients with, or at risk of, type 2 diabetes mellitus: A systematic review and meta-analysis. Physiol Behav. 2020;215:112786.

27. Jaksch W, Dejaco C, Schirmer M. 4 years after withdrawal of rofecoxib: where do we stand today? Rheumatol Int. 2008;28(12):1187-95.

28. Jeon SR, Nam D, Kim TH. Dropouts in randomized clinical trials of Korean medicine interventions: a systematic review and meta-analysis. Trials. 2021;22(1):176.

29. Johnco C, McGuire JF, Roper T, Storch EA. A meta-analysis of dropout rates from exposure with response prevention and pharmacological treatment for youth with obsessive compulsive disorder. Depress Anxiety. 2020;37(5):407-17.

30. Kan C, Eid L, Treasure J, Himmerich H. A Meta-Analysis of Dropout and Metabolic Effects of Antipsychotics in Anorexia Nervosa. Front Psychiatry. 2020;11:208.

31. Karyotaki E, Kleiboer A, Smit F, Turner DT, Pastor AM, Andersson G, et al. Predictors of treatment dropout in self-guided web-based interventions for depression: an 'individual patient data' meta-analysis. Psychol Med. 2015;45(13):2717-26.

32. Knippschild S, Hirsch J, Krummenauer F. [Metaanalysis to estimate the expected drop out-rates reported in clinical trials on cataract surgery]. Klin Monbl Augenheilkd. 2014;231(2):151-7.

33. Kuszmaul AK, Palmer EC, Frederick EK. Lofexidine versus clonidine for mitigation of opioid withdrawal symptoms: A systematic review. J Am Pharm Assoc (2003). 2020;60(1):145-52.

34. Lappan SN, Brown AW, Hendricks PS. Dropout rates of in-person psychosocial substance use disorder treatments: a systematic review and meta-analysis. Addiction. 2020;115(2):201-17.

35. Lewis C, Roberts NP, Gibson S, Bisson JI. Dropout from psychological therapies for post-traumatic stress disorder (PTSD) in adults: systematic review and meta-analysis. Eur J Psychotraumatol. 2020;11(1):1709709.

36. Linardon J, Fitzsimmons-Craft EE, Brennan L, Barillaro M, Wilfley DE. Dropout from interpersonal psychotherapy for mental health disorders: A systematic review and meta-analysis. Psychother Res. 2019;29(7):870-81.

37. Linardon J, Hindle A, Brennan L. Dropout from cognitive-behavioral therapy for eating disorders: A meta-analysis of randomized, controlled trials. Int J Eat Disord. 2018;51(5):381-91.

38. Makatsori M, Scadding GW, Lombardo C, Bisoffi G, Ridolo E, Durham SR, et al. Dropouts in sublingual allergen immunotherapy trials - a systematic review. Allergy. 2014;69(5):571-80.

39. Martin JL, Pérez V, Sacristán M, Rodríguez-Artalejo F, Martínez C, Alvarez E. Meta-analysis of drop-out rates in randomised clinical trials, comparing typical and atypical antipsychotics in the treatment of schizophrenia. Eur Psychiatry. 2006;21(1):11-20.

40. Matsusaki A, Kaneko M, Narukawa M. Meta-analysis of Dropout Rates in Placebo-Controlled Randomized Clinical Trials of Atypical Antipsychotics Assessed by PANSS. Clin Drug Investig. 2019;39(10):917-26.

41. McLean C, Tapsell L, Grafenauer S, McMahon AT. Systematic review of nutritional interventions for people admitted to hospital for alcohol withdrawal. Nutr Diet. 2020;77(1):76-89.

42. Melville KM, Casey LM, Kavanagh DJ. Psychological treatment dropout among pathological gamblers. Clin Psychol Rev. 2007;27(8):944-58.

43. Meske DS, Lawal OD, Elder H, Langberg V, Paillard F, Katz N. Efficacy of opioids versus placebo in chronic pain: a systematic review and meta-analysis of enriched enrollment randomized withdrawal trials. J Pain Res. 2018;11:923-34.

44. Meyerowitz-Katz G, Ravi S, Arnolda L, Feng X, Maberly G, Astell-Burt T. Rates of Attrition and Dropout in App-Based Interventions for Chronic Disease: Systematic Review and Meta-Analysis. J Med Internet Res. 2020;22(9):e20283.

45. Montgomery SA, Kasper S. Side effects, dropouts from treatment and cost consequences. Int Clin Psychopharmacol. 1998;13 Suppl 2:S1-5.

46. Moroshko I, Brennan L, O'Brien P. Predictors of dropout in weight loss interventions: a systematic review of the literature. Obes Rev. 2011;12(11):912-34.

47. Mutsaerts MA, Kuchenbecker WK, Mol BW, Land JA, Hoek A. Dropout is a problem in lifestyle intervention programs for overweight and obese infertile women: a systematic review. Hum Reprod. 2013;28(4):979-86.

48. Møllerløkken NE, Lorås H, Pedersen AV. A SYSTEMATIC REVIEW AND META-ANALYSIS OF DROPOUT RATES IN YOUTH SOCCER. Percept Mot Skills. 2015;121(3):913-22.

49. Nelson M, Reid C, Krum H, McNeil J. A systematic review of predictors of maintenance of normotension after withdrawal of antihypertensive drugs. Am J Hypertens. 2001;14(2):98-105.

50. Ong CW, Clyde JW, Bluett EJ, Levin ME, Twohig MP. Dropout rates in exposure with response prevention for obsessive-compulsive disorder: What do the data really say? J Anxiety Disord. 2016;40:8-17.

51. Oosterhaven J, Wittink H, Mollema J, Kruitwagen C, Devillé W. Predictors of dropout in interdisciplinary chronic pain management programmes: A systematic review. J Rehabil Med. 2019;51(1):2-10.

52. Pfund RA, Peter SC, McAfee NW, Ginley MK, Whelan JP, Meyers AW. Dropout from face-to-face, multi-session psychological treatments for problem and disordered gambling: A systematic review and meta-analysis. Psychol Addict Behav. 2021;35(8):901-13.

53. Pozza A, Dèttore D. Drop-out and efficacy of group versus individual cognitive behavioural therapy: What works best for Obsessive-Compulsive Disorder? A systematic review and meta-analysis of direct comparisons. Psychiatry Res. 2017;258:24-36.

54. Rabinowitz J, Davidov O. A composite approach that includes dropout rates when analyzing efficacy data in clinical trials of antipsychotic medications. Schizophr Bull. 2008;34(6):1145-50.

55. Rehman Y, Ferguson H, Bozek A, Blair J, Allison A, Johnston R. Dropout associated with osteopathic manual treatment for chronic noncancerous pain in randomized controlled trials. J Osteopath Med. 2021;121(4):417-28.

56. Reljic D, Lampe D, Wolf F, Zopf Y, Herrmann HJ, Fischer J. Prevalence and predictors of dropout from high-intensity interval training in sedentary individuals: A meta-analysis. Scand J Med Sci Sports. 2019;29(9):1288-304.

57. Resurrección DM, Moreno-Peral P, Gómez-Herranz M, Rubio-Valera M, Pastor L, Caldas de Almeida JM, et al. Factors associated with non-participation in and dropout from cardiac rehabilitation programmes: a systematic review of prospective cohort studies. Eur J Cardiovasc Nurs. 2019;18(1):38-47.

58. Resurrección DM, Motrico E, Rigabert A, Rubio-Valera M, Conejo-Cerón S, Pastor L, et al. Barriers for Nonparticipation and Dropout of Women in Cardiac Rehabilitation Programs: A Systematic Review. J Womens Health (Larchmt). 2017;26(8):849-59.

59. Rohden AI, Benchaya MC, Camargo RS, Moreira TC, Barros HMT, Ferigolo M. Dropout Prevalence and Associated Factors in Randomized Clinical Trials of Adolescents Treated for Depression: Systematic Review and Meta-analysis. Clin Ther. 2017;39(5):971-92.e4.

60. Rutherford BR, Sneed JR, Roose SP. Does differential drop-out explain the influence of study design on antidepressant response? A meta-analysis. J Affect Disord. 2012;140(1):57-65.

61. Sciarrino NA, Bartlett BA, Smith LJ, Martin CE, Williams W. Factors contributing to PTSD treatment dropout in veterans returning from the wars in Iraq and Afghanistan: A systematic review. Psychol Serv. 2022;19(1):183-200.

62. Sharma T, Guski LS, Freund N, Meng DM, Gøtzsche PC. Drop-out rates in placebo-controlled trials of antidepressant drugs: A systematic review and meta-analysis based on clinical study reports. Int J Risk Saf Med. 2019;30(4):217-32.

63. Simmons C, Meiser-Stedman R, Baily H, Beazley P. A meta-analysis of dropout from evidence-based psychological treatment for post-traumatic stress disorder (PTSD) in children and young people. Eur J Psychotraumatol. 2021;12(1):1947570.

64. Srisurapanont M, Likhitsathian S, Suttajit S, Maneeton N, Maneeton B, Oon-Arom A, et al. Efficacy and dropout rates of antipsychotic medications for methamphetamine psychosis: A systematic review and network meta-analysis. Drug Alcohol Depend. 2021;219:108467.

65. Steins Bisschop CN, Courneya KS, Velthuis MJ, Monninkhof EM, Jones LW, Friedenreich C, et al. Control group design, contamination and drop-out in exercise oncology trials: a systematic review. PLoS One. 2015;10(3):e0120996.

66. Stubbs B, Vancampfort D, Rosenbaum S, Ward PB, Richards J, Soundy A, et al. Dropout from exercise randomized controlled trials among people with depression: A meta-analysis and meta regression. J Affect Disord. 2016;190:457-66.

67. Tanner-Smith EE, Wilson SJ. A meta-analysis of the effects of dropout prevention programs on school absenteeism. Prev Sci. 2013;14(5):468-78.

68. Torous J, Lipschitz J, Ng M, Firth J. Dropout rates in clinical trials of smartphone apps for depressive symptoms: A systematic review and meta-analysis. J Affect Disord. 2020;263:413-9.

69. Townsend L, Flisher AJ, King G. A systematic review of the relationship between high school dropout and substance use. Clin Child Fam Psychol Rev. 2007;10(4):295-317.

70. Vancampfort D, Mugisha J, Richards J, De Hert M, Lazzarotto AR, Schuch FB, et al. Dropout from physical activity interventions in people living with HIV: a systematic review and meta-analysis. AIDS Care. 2017;29(5):636-43.

71. Vancampfort D, Sánchez CPR, Hallgren M, Schuch F, Firth J, Rosenbaum S, et al. Dropout from exercise randomized controlled trials among people with anxiety and stress-related disorders: A meta-analysis and meta-regression. J Affect Disord. 2021;282:996-1004.

72. Windle E, Tee H, Sabitova A, Jovanovic N, Priebe S, Carr C. Association of Patient Treatment Preference With Dropout and Clinical Outcomes in Adult Psychosocial Mental Health Interventions: A Systematic Review and Meta-analysis. JAMA Psychiatry. 2020;77(3):294-302.

73. Wright I, Mughal F, Bowers G, Meiser-Stedman R. Dropout from randomised controlled trials of psychological treatments for depression in children and youth: a systematic review and meta-analyses. J Affect Disord. 2021;281:880-90.

74. Zagmutt FJ, Tarrants ML. Indirect comparisons of adverse events and dropout rates in early Parkinson's disease trials of pramipexole, ropinirole, and rasagiline. Int J Neurosci. 2012;122(7):345-53.

75. Zhong X, Zhang T, Liu Y, Wei X, Zhang X, Qin Y, et al. Effects of three injectable antidiabetic agents on glycaemic control, weight change and drop-out in type 2 diabetes suboptimally controlled with metformin and/or a sulfonylurea: A network meta-analysis. Diabetes Res Clin Pract. 2015;109(3):451-60.

76. Zhou Y, Sun L, Wang Y, Wu L, Sun Z, Zhang F, et al. Developments of prolonged exposure in treatment effect of post-traumatic stress disorder and controlling dropout rate: A meta-analytic review. Clin Psychol Psychother. 2020;27(4):449-62.

Appendix C. Pragmatic search of reviews assessing relevant intervention trials.

The pragmatic search was conducted in PubMed [12] on 14. February 2022 using the following "Boolean terms":

Conditions: crohn*[tiab] *OR* colitis[tiab] *OR* IBD[tiab]

Interventions: nutri*[tiab] *OR* diet*[tiab] *OR* elimination*[tiab] *OR* "diet therapy" [tiab] *OR* fibre[tiab] *OR* fiber[tiab] *OR* protein*[tiab] *OR* omega*[tiab] *OR* carbo*[tiab]

Design/Methodology: "Cochrane Database Syst Rev"[jour] *OR* meta-analysis[pt] *OR* "systematic review"[pt] *OR* meta-analys*[pt] *OR* meta-analys*[ti] *OR* metaanalys*[ti] *OR* meta-regress*[tiab] *OR* metaregress*[tiab]

Additionally, we searched Cochrane Library on 19. May 2022 using 'inflammatory bowel disease' in title, abstract and keywords and 'diet*' or 'nutrition*' in record title.

This search, after removal of duplicates, resulted in 199 articles of which 33 systematic reviews (table 1 below) addressed the effects of diet on symptoms or disease remission in IBD as judged by the title and abstract. Of these 31 studies: 13 assessed unspecified diets on disease activity (2, 7, 12, 13, 17, 22, 28, 31, 32), microbiome alterations (3), IBD symptoms (1, 17, 19, 25), or operative outcomes (11), 16 assessed seven different specified dietary components on disease activity (i.e. fatty acids (4, 5, 10, 24, 27, 29, 30, 32), curcumin (6, 14, 16), starch (9), glutamine (21), fibre (23), herbal therapies (26), and propolis (20)), and two assessed the effect of low FODMAP diets on IBD symptoms (8, 15). Excluded reviews included those, which assessed risk factors for developing new cases of CID, effects of non-dietary interventions, or non-systematic reviews.

Table 1. Overview of potentially relevant systematic reviews

Ref.	Publications presented in order of publication date
1	Sinopoulou, V, et al. (2021). "Interventions for the management of abdominal pain in Crohn's disease and inflammatory bowel disease." Cochrane Database Syst Rev. 11(11).
2	Barros VJDS, et al. (2021). "Effect of dietary interventions on inflammatory biomarkers of inflammatory bowel diseases: A systematic review of clinical trials." Nutrition 91-92.
3	Wagenaar CA, et al. (2021). "The Effect of Dietary Interventions on Chronic Inflammatory Diseases in Relation to the Microbiome: A Systematic Review." Nutrients 13(9).
4	Ajabnoor SM, et al. (2021). "Long-term effects of increasing omega-3, omega-6 and total polyunsaturated fats on inflammatory bowel disease and markers of inflammation: a systematic review and metaanalysis of randomized controlled trials. Eur J Nutr. 60(5).
5	Basson AR, et al. (2021). "Regulation of Intestinal Inflammation by Dietary Fats." Front Immunol. 11.
6	Atefi M, et al. (2021). "A Systematic Review of the Clinical Use of Curcumin for the Management of Gastrointestinal Diseases." Adv Exp Med Biol. 1291(295-326).
7	Comeche JM, et al. (2020). "Predefined Diets in Patients with Inflammatory Bowel Disease: Systematic Review and Meta-Analysis." Nutrients. 13(1).
8	Grammatikopoulou MG, et al. (2020). Low FODMAP Diet for Functional Gastrointestinal Symptoms in Quiescent Inflammatory Bowel Disease: A Systematic Review of Randomized Controlled Trials. Nutrients. 12(12).
9	Montroy J, et al. (2020): The effects of resistant starches on inflammatory bowel disease in preclinical and clinical settings: a systematic review and meta-analysis. BMC Gastroenterol. 20(1).

10	Schwermer M, et al. (2020): Complementary, alternative, integrative and dietary therapies for children with Crohn's disease - A systematic review. Complement Ther Med. 52.
11	Adamina M, et al. (2020): Perioperative Dietary Therapy in Inflammatory Bowel Disease. J Crohns Colitis. 14(4)
12	McVeigh L and Payne A (2020): Inducing remission in paediatric Crohn's disease using nutritional therapies - A systematic review. J Hum Nutr Diet. 33(2).
13	Limketkai BN, et al. (2019): Dietary interventions for induction and maintenance of remission in inflammatory bowel disease. Cochrane Database of Systematic Reviews 2:CD012839. DOI: 10.1002/14651858.CD012839.pub2.
14	Grammatikopoulou MG, et al. (2018): Oral Adjuvant Curcumin Therapy for Attaining Clinical Remission in Ulcerative Colitis: A Systematic Review and Meta-Analysis of Randomized Controlled Trials. Nutrients. 10(11).
15	Zhan YL, et al. (2018): Is a low FODMAP diet beneficial for patients with inflammatory bowel disease? A metaanalysis and systematic review. Clin Nutr. 37(1).
16	Schneider A, et al. (2017): Comparison of remicade to curcumin for the treatment of Crohn's disease: A systematic review. Complement Ther Med. 33:32-38.
17	Norton C, et al. (2017): Systematic review: interventions for abdominal pain management in inflammatory bowel disease. Aliment Pharmacol Ther. 46(2).
18	Forbes A, et al. (2017): ESPEN guideline: Clinical nutrition in inflammatory bowel disease. Clin Nutr. 36(2).
19	Charlebois A, et al. (2016): The Impact of Dietary Interventions on the Symptoms of Inflammatory Bowel Disease: A Systematic Review. Crit Rev Food Sci Nutr. 56(8).
20	Penagini F, et al. (2016): Nutrition in Pediatric Inflammatory Bowel Disease: From Etiology to Treatment. A Systematic Review. Nutrients. 8(6).
21	Akobeng AK, et al. (2016): Glutamine for induction of remission in Crohn's disease. Cochrane Database Syst Rev. 2.
22	Tsertsvadze A, et al. (2015): Clinical effectiveness and cost-effectiveness of elemental nutrition for the maintenance of remission in Crohn's disease: a systematic review and meta-analysis. Health Technol Assess. 19(26).
23	Wedlake L, et al. (2014): Fiber in the treatment and maintenance of inflammatory bowel disease: a systematic review of randomized controlled trials. Inflamm Bowel Dis. 20(3).
24	Lev-Tzion R, et al. (2014): Omega 3 fatty acids (fish oil) for maintenance of remission in Crohn's disease. Cochrane Database Syst Rev. 2.
25	Henson CC, et al. (2013): Nutritional interventions for reducing gastrointestinal toxicity in adults undergoing radical pelvic radiotherapy. Cochrane Database of Systematic Reviews. 11.:CD009896. DOI: 10.1002/14651858.CD009896.pub2.
26	Ng SC, et al. (2013): Systematic review: the efficacy of herbal therapy in inflammatory bowel disease. Aliment Pharmacol Ther. 38(8).
27	Swan K and Allen PJ (2013): Omega-3 fatty acid for the treatment and remission of Crohn's disease. J Complement Integr Med. 10.
28	Sung MK and Park MY (2013): Nutritional modulators of ulcerative colitis: clinical efficacies and mechanistic view. World J Gastroenterol. 19(7).
29	Cabré E, et al. (2012): Omega-3 fatty acids and inflammatory bowel diseases - a systematic review. Br J Nutr. 107 Suppl 2.
30	Turner D, et al. (2011): Maintenance of remission in inflammatory bowel disease using omega-3 fatty acids (fish oil): a systematic review and meta-analyses. Inflamm Bowel Dis. 17(1).
31	Pimentel-Nunes P, et al. (2009): Systematic review on drug and diet-induced endoscopic remission in Crohn's disease. Eur J Gastroenterol Hepatol. 21(5).
32	Turner D, et al. (2009): Omega 3 fatty acids (fish oil) for maintenance of remission in Crohn's disease. Cochrane Database Syst Rev. 1.
33	Day AS, et al. (2008): Systematic review: nutritional therapy in paediatric Crohn's disease. Aliment Pharmacol Ther. 27(4).

Appendix D. PRISMA-P checklist.

PRISMA-P (Preferred Reporting Items for Systematic review and Meta-Analysis Protocols) 2015 checklist: recommended items to address in a systematic review protocol*

Section and topic	Item No	Checklist item	Pg.
**ADMINISTRATIVE INFORMATION**			
Title:			
Identification	1a	Identify the report as a protocol of a systematic review	1
Update	1b	If the protocol is for an update of a previous systematic review, identify as such	-
Registration	2	If registered, provide the name of the registry (such as PROSPERO) and registration number	1
Authors:			
Contact	3a	Provide name, institutional affiliation, e-mail address of all protocol authors; provide physical mailing address of corresponding author	1
Contributions	3b	Describe contributions of protocol authors and identify the guarantor of the review	1
Amendments	4	If the protocol represents an amendment of a previously completed or published protocol, identify as such and list changes; otherwise, state plan for documenting important protocol amendments	-
Support:			
Sources	5a	Indicate sources of financial or other support for the review	1
Sponsor	5b	Provide name for the review funder and/or sponsor	1
Role of sponsor or funder	5c	Describe roles of funder(s), sponsor(s), and/or institution(s), if any, in developing the protocol	1
**INTRODUCTION**			
Rationale	6	Describe the rationale for the review in the context of what is already known	2-3
Objectives	7	Provide an explicit statement of the question(s) the review will address with reference to participants, interventions, comparators, and outcomes (PICO)	4
**METHODS**			
Eligibility criteria	8	Specify the study characteristics (such as PICO, study design, setting, time frame) and report characteristics (such as years considered, language, publication status) to be used as criteria for eligibility for the review	5

Information sources	9	Describe all intended information sources (such as electronic databases, contact with study authors, trial registers or other grey literature sources) with planned dates of coverage	5
Search strategy	10	Present draft of search strategy to be used for at least one electronic database, including planned limits, such that it could be repeated	5-6 + 28
Study records:			
Data management	11a	Describe the mechanism(s) that will be used to manage records and data throughout the review	6
Selection process	11b	State the process that will be used for selecting studies (such as two independent reviewers) through each phase of the review (that is, screening, eligibility and inclusion in meta-analysis)	6
Data collection process	11c	Describe planned method of extracting data from reports (such as piloting forms, done independently, in duplicate), any processes for obtaining and confirming data from investigators	6-7
Data items	12	List and define all variables for which data will be sought (such as PICO items, funding sources), any pre-planned data assumptions and simplifications	7
Outcomes and prioritization	13	List and define all outcomes for which data will be sought, including prioritization of main and additional outcomes, with rationale	7
Risk of bias in individual studies	14	Describe anticipated methods for assessing risk of bias of individual studies, including whether this will be done at the outcome or study level, or both; state how this information will be used in data synthesis	7
Data synthesis	15a	Describe criteria under which study data will be quantitatively synthesised	10
	15b	If data are appropriate for quantitative synthesis, describe planned summary measures, methods of handling data and methods of combining data from studies, including any planned exploration of consistency (such as I^2, Kendall's τ)	10
	15c	Describe any proposed additional analyses (such as sensitivity or subgroup analyses, meta-regression)	8-9
	15d	If quantitative synthesis is not appropriate, describe the type of summary planned	-
Meta-bias(es)	16	Specify any planned assessment of meta-bias(es) (such as publication bias across studies, selective reporting within studies)	7-8
Confidence in cumulative evidence	17	Describe how the strength of the body of evidence will be assessed (such as GRADE)	10

*** It is strongly recommended that this checklist be read in conjunction with the PRISMA-P Explanation and Elaboration (cite when available) for important clarification on the items. Amendments to a review protocol should be tracked and dated.** **The copyright for PRISMA-P (including checklist) is held by the PRISMA-P Group and is distributed under a Creative Commons Attribution Licence 4.0.**

From: Shamseer L, Moher D, Clarke M, Ghersi D, Liberati A, Petticrew M, Shekelle P, Stewart L, PRISMA-P Group. Preferred reporting items for systematic review and meta-analysis protocols (PRISMA-P) 2015: elaboration and explanation. BMJ. 2015 Jan 2;349(jan02 1):g7647.

Appendix E. MEDLINE search strategy.

1.	exp Inflammatory Bowel Diseases/
2.	exp Colitis, Ulcerative/ or Colitis/
3.	exp Crohn Disease/
4.	exp Proctitis/
5.	crohn*.mp.
6.	(ulcerative adj colitis).mp.
7.	"inflammatory bowel disease*".mp.
8.	IBD.mp.
9.	1 or 2 or 3 or 4 or 5 or 6 or 7 or 8
10.	Clinical Trials as Topic/
11.	Randomized controlled trial.pt.
12.	Controlled clinical trial.pt.
13.	randomized.ab.
14.	placebo.ab.
15.	randomly.ab.
16.	trial.ti.
17.	10 or 11 or 12 or 13 or 14 or 15 or 16
18.	exp animals/ not humans.sh.
19.	17 not 18
20.	exp Diet/
21.	exp Diet Therapy/
22.	exp Enteral Nutrition/
23.	regimen.ti,ab.
24.	nutrition.ti,ab.
25.	eliminate*.ti,ab.
26.	(food* or fodmap* or gluten* or keto* or pal$eo* or vegetarian* or vegan*).ti,ab.
27.	(oligosaccharide* or oligofructose* or monosaccharide* or disaccharide* or fructooligosaccharide*).ti,ab.
28.	(carb* or sugar* or fiber* or fibre* or omega* or fatty acid* or high-fat* or high fat* or protein* or high-protein* or high protein*).ti,ab.
29.	(fruit* or vegetable* or dairy* or fish* or meat).ti,ab.
30.	sodium*.ti,ab.
31.	(macronutirent* or macro nutrient* or macro-nutrient*).ti,ab.
32.	(micronutrient* or micro nutrient* or micro-nutrient*).ti,ab.
33.	fast*.ti,ab.
34.	(diet* or diet therapy* or enteral nutrition*).ti,ab.
35.	20 or 21 or 22 or 23 or 24 or 25 or 26 or 27 or 28 or 29 or 30 or 31 or 32 or 33 or 34
36.	9 and 19 and 35

References

1. Tatsioni, A. and J.P.A. Ioannidis, *Meta-research: bird's eye views of primary care research.* Family Practice, 2020. **37**(3): p. 287-289.
2. Ioannidis, J.P.A., et al., *Meta-research: Evaluation and Improvement of Research Methods and Practices.* PLoS biology, 2015. **13**(10): p. e1002264-e1002264.
3. Lawson, D.O., et al., *Reporting of methodological studies in health research: a protocol for the development of the MethodologIcal STudy reportIng Checklist (MISTIC).* BMJ Open, 2020. **10**(12): p. e040478.
4. McGee, R.G. and A.C. Dawson, *Fake news and fake research: Why meta-research matters more than ever.* Journal of paediatrics and child health, 2020. **56**(12): p. 1868-1871.
5. Nielsen, O.H. and M.A. Ainsworth, *Tumor necrosis factor inhibitors for inflammatory bowel disease.* N Engl J Med, 2013. **369**(8): p. 754-62.
6. Baumgart, D.C. and W.J. Sandborn, *Inflammatory bowel disease: clinical aspects and established and evolving therapies.* Lancet, 2007. **369**(9573): p. 1641-57.
7. Crooks, B., et al., *The dietary practices and beliefs of people living with older-onset inflammatory bowel disease.* Eur J Gastroenterol Hepatol, 2021.
8. Crooks, B., et al., *The dietary practices and beliefs of people living with inactive ulcerative colitis.* Eur J Gastroenterol Hepatol, 2021. **33**(3): p. 372-379.
9. RUBIN, D.B., *Inference and missing data.* Biometrika, 1976. **63**(3): p. 581-592.
10. O'Neill, R.T. and R. Temple, *The prevention and treatment of missing data in clinical trials: an FDA perspective on the importance of dealing with it.* Clin Pharmacol Ther, 2012. **91**(3): p. 550-4.
11. Lund, H., et al., *Evidence-Based Research Series-Paper 2 : Using an Evidence-Based Research approach before a new study is conducted to ensure value.* J Clin Epidemiol, 2021. **129**: p. 158-166.
12. Lund, H., C. Juhl, and R. Christensen, *Systematic reviews and research waste.* Lancet, 2016. **387**(10014): p. 123-4.
13. Lund, H., et al., *Towards evidence based research.* Bmj, 2016. **355**: p. i5440.
14. Vancampfort, D., et al., *Dropout from physical activity interventions in people living with HIV: a systematic review and meta-analysis.* AIDS Care, 2017. **29**(5): p. 636-643.
15. Jabardo-Camprubí, G., et al., *Drop-out ratio between moderate to high-intensity physical exercise treatment by patients with, or at risk of, type 2 diabetes mellitus: A systematic review and meta-analysis.* Physiol Behav, 2020. **215**: p. 112786.
16. Dennett, R., et al., *Adherence and drop-out in randomized controlled trials of exercise interventions in people with multiple sclerosis: A systematic review and meta-analyses.* Mult Scler Relat Disord, 2020. **43**: p. 102169.
17. Meyerowitz-Katz, G., et al., *Rates of Attrition and Dropout in App-Based Interventions for Chronic Disease: Systematic Review and Meta-Analysis.* J Med Internet Res, 2020. **22**(9): p. e20283.
18. Steins Bisschop, C.N., et al., *Control group design, contamination and drop-out in exercise oncology trials: a systematic review.* PLoS One, 2015. **10**(3): p. e0120996.
19. Cramer, H., et al., *A Systematic Review and Meta-Analysis Estimating the Expected Dropout Rates in Randomized Controlled Trials on Yoga Interventions.* Evid Based Complement Alternat Med, 2016. **2016**: p. 5859729.
20. Zhong, X., et al., *Effects of three injectable antidiabetic agents on glycaemic control, weight change and drop-out in type 2 diabetes suboptimally controlled with metformin and/or a sulfonylurea: A network meta-analysis.* Diabetes Res Clin Pract, 2015. **109**(3): p. 451-60.
21. Makatsori, M., et al., *Dropouts in sublingual allergen immunotherapy trials - a systematic review.* Allergy, 2014. **69**(5): p. 571-80.
22. Corona, G., et al., *First-generation phosphodiesterase type 5 inhibitors dropout: a comprehensive review and meta-analysis.* Andrology, 2016. **4**(6): p. 1002-1009.
23. Jeon, S.R., D. Nam, and T.H. Kim, *Dropouts in randomized clinical trials of Korean medicine interventions: a systematic review and meta-analysis.* Trials, 2021. **22**(1): p. 176.

24. Shamseer, L., et al., *Preferred reporting items for systematic review and meta-analysis protocols (PRISMA-P) 2015: elaboration and explanation.* BMJ : British Medical Journal, 2015. **349**: p. g7647.
25. Hunter, K.E., et al., *Searching clinical trials registers: guide for systematic reviewers.* BMJ, 2022. **377**: p. e068791.
26. Lefebvre C, Glanville J, Briscoe S, Littlewood A, Marshall C, Metzendorf M-I, Noel-Storr A, Rader T, Shokraneh F, Thomas J, Wieland LS. Technical Supplement to Chapter 4: Searching for and selecting studies. In: Higgins JPT, Thomas J, Chandler J, Cumpston MS, Li T, Page MJ, Welch VA (eds). Cochrane Handbook for Systematic Reviews of Interventions Version 6. Cochrane, 2019. Available from: www.training.cochrane.org/handbook.
27. Lee, S., et al., *The impact of surgical therapies for inflammatory bowel disease on female fertility.* Cochrane Database of Systematic Reviews, 2019(7).
28. Limketkai, B.N., et al., *Dietary interventions for induction and maintenance of remission in inflammatory bowel disease.* Cochrane Database Syst Rev, 2019. **2**(2): p. Cd012839.
29. McGowan, J., et al., *PRESS Peer Review of Electronic Search Strategies: 2015 Guideline Statement.* J Clin Epidemiol, 2016. **75**: p. 40-6.
30. McKeown, S. and Z.M. Mir, *Considerations for conducting systematic reviews: evaluating the performance of different methods for de-duplicating references.* Syst Rev, 2021. **10**(1): p. 38.
31. Higgins, J.P.T., et al., *The Cochrane Collaboration's tool for assessing risk of bias in randomised trials.* BMJ, 2011. **343**: p. d5928.
32. Murimi, M.W., et al., *Factors Influencing Efficacy of Nutrition Education Interventions: A Systematic Review.* J Nutr Educ Behav, 2017. **49**(2): p. 142-165.e1.
33. Murimi, M.W., et al., *Factors that contribute to effective online nutrition education interventions: a systematic review.* Nutr Rev, 2019. **77**(10): p. 663-690.
34. Sterne, J.A.C., et al., *Recommendations for examining and interpreting funnel plot asymmetry in meta-analyses of randomised controlled trials.* BMJ, 2011. **343**: p. d4002.
35. Higgins, J.P., et al., *Measuring inconsistency in meta-analyses.* Bmj, 2003. **327**(7414): p. 557-60.
36. Christensen, R. and D.B. Berthelsen, *Controversy and Debate on Meta-epidemiology. Paper 3: Causal inference from meta-epidemiology: a reasonable goal, or wishful thinking?* J Clin Epidemiol, 2020. **123**: p. 131-132.

Supplementary material to:

Study Design Complexity and Participant Completion in Dietary Trials for Inflammatory Bowel Disease: A Systematic Review and Meta-Research Study

First Author: Laura Gregersen

Supplementary material S2: PRISMA 2020 Checklist

PRISMA

PRISMA 2020 Checklist

Section and Topic	Item #	Checklist item	Location where item is reported
**TITLE**			
Title	1	Identify the report as a systematic review.	Title
**ABSTRACT**			
Abstract	2	See the PRISMA 2020 for Abstracts checklist.	Pg. 3
**INTRODUCTION**			
Rationale	3	Describe the rationale for the review in the context of existing knowledge.	Section 1
Objectives	4	Provide an explicit statement of the objective(s) or question(s) the review addresses.	Section 1
**METHODS**			
Eligibility criteria	5	Specify the inclusion and exclusion criteria for the review and how studies were grouped for the syntheses.	Section 2.2.
Information sources	6	Specify all databases, registers, websites, organisations, reference lists and other sources searched or consulted to identify studies. Specify the date when each source was last searched or consulted.	Section 2.1.
Search strategy	7	Present the full search strategies for all databases, registers and websites, including any filters and limits used.	Suppl. S1
Selection process	8	Specify the methods used to decide whether a study met the inclusion criteria of the review, including how many reviewers screened each record and each report retrieved, whether they worked independently, and if applicable, details of automation tools used in the process.	Section 2.1.
Data collection process	9	Specify the methods used to collect data from reports, including how many reviewers collected data from each report, whether they worked independently, any processes for obtaining or confirming data from study investigators, and if applicable, details of automation tools used in the process.	Section 2.3.
Data items	10a	List and define all outcomes for which data were sought. Specify whether all results that were compatible with each outcome domain in each study were sought (e.g. for all measures, time points, analyses), and if not, the methods used to decide which results to collect.	Section 2.4.
	10b	List and define all other variables for which data were sought (e.g. participant and intervention characteristics, funding sources). Describe any assumptions made about any missing or unclear information.	Section 2.5.
Study risk of bias assessment	11	Specify the methods used to assess risk of bias in the included studies, including details of the tool(s) used, how many reviewers assessed each study and whether they worked independently, and if applicable, details of automation tools used in the process.	Section 2.3.
Effect measures	12	Specify for each outcome the effect measure(s) (e.g. risk ratio, mean difference) used in the synthesis or presentation of results.	Section 2.4.
Synthesis methods	13a	Describe the processes used to decide which studies were eligible for each synthesis (e.g. tabulating the study intervention characteristics and comparing against the planned groups for each synthesis (item #5)).	Section 2.1.
	13b	Describe any methods required to prepare the data for presentation or synthesis, such as handling of missing summary statistics, or data conversions.	Section 3.1.
	13c	Describe any methods used to tabulate or visually display results of individual studies and syntheses.	Section 3
	13d	Describe any methods used to synthesize results and provide a rationale for the choice(s). If meta-analysis was performed, describe the model(s), method(s) to identify the presence and extent of statistical heterogeneity, and software package(s) used.	Section 3
	13e	Describe any methods used to explore possible causes of heterogeneity among study results (e.g. subgroup analysis, meta-regression).	Section 3
	13f	Describe any sensitivity analyses conducted to assess robustness of the synthesized results.	Section 3.2
Reporting bias assessment	14	Describe any methods used to assess risk of bias due to missing results in a synthesis (arising from reporting biases).	Section 2.3.
Certainty assessment	15	Describe any methods used to assess certainty (or confidence) in the body of evidence for an outcome.	Section 3

PRISMA 2020 Checklist

Section and Topic	Item #	Checklist item	Location where item is reported
**RESULTS**			
Study selection	16a	Describe the results of the search and selection process, from the number of records identified in the search to the number of studies included in the review, ideally using a flow diagram.	Section 4
	16b	Cite studies that might appear to meet the inclusion criteria, but which were excluded, and explain why they were excluded.	N/A
Study characteristics	17	Cite each included study and present its characteristics.	Table 1 + Suppl. S3
Risk of bias in studies	18	Present assessments of risk of bias for each included study.	Suppl. S4
Results of individual studies	19	For all outcomes, present, for each study: (a) summary statistics for each group (where appropriate) and (b) an effect estimate and its precision (e.g. confidence/credible interval), ideally using structured tables or plots.	Table 2+3
Results of syntheses	20a	For each synthesis, briefly summarise the characteristics and risk of bias among contributing studies.	Section 4-4.7. + Suppl. S4
	20b	Present results of all statistical syntheses conducted. If meta-analysis was done, present for each the summary estimate and its precision (e.g. confidence/credible interval) and measures of statistical heterogeneity. If comparing groups, describe the direction of the effect.	Fig. 1 + table 2+3
	20c	Present results of all investigations of possible causes of heterogeneity among study results.	Table 2+3
	20d	Present results of all sensitivity analyses conducted to assess the robustness of the synthesized results.	Section 4.5 + table 4
Reporting biases	21	Present assessments of risk of bias due to missing results (arising from reporting biases) for each synthesis assessed.	Suppl. S4
Certainty of evidence	22	Present assessments of certainty (or confidence) in the body of evidence for each outcome assessed.	Table 2+3
**DISCUSSION**			
Discussion	23a	Provide a general interpretation of the results in the context of other evidence.	Section 5-5.4.
	23b	Discuss any limitations of the evidence included in the review.	Section 5.6.
	23c	Discuss any limitations of the review processes used.	Section 5.6.
	23d	Discuss implications of the results for practice, policy, and future research.	Section 5.5.
**OTHER INFORMATION**			
Registration and protocol	24a	Provide registration information for the review, including register name and registration number, or state that the review was not registered.	Section 2
	24b	Indicate where the review protocol can be accessed, or state that a protocol was not prepared.	Section 2
	24c	Describe and explain any amendments to information provided at registration or in the protocol.	Section 2.5. + 3.2. + 4.3.
Support	25	Describe sources of financial or non-financial support for the review, and the role of the funders or sponsors in the review.	Page 2
Competing interests	26	Declare any competing interests of review authors.	Page 2
Availability of data, code and	27	Report which of the following are publicly available and where they can be found: template data collection forms; data extracted from included studies; data used for all analyses; analytic code; any other materials used in the review.	Page 2

Section and Topic	Item #	Checklist item	Location where item is reported
other materials			

From: Page MJ, McKenzie JE, Bossuyt PM, Boutron I, Hoffmann TC, Mulrow CD, et al. The PRISMA 2020 statement: an updated guideline for reporting systematic reviews. BMJ 2021;372:n71. doi: 10.1136/bmj.n71.
This work is licensed under CC BY 4.0. To view a copy of this license, visit https://creativecommons.org/licenses/by/4.0/

Supplementary material to:

Study Design Complexity and Participant Completion in Dietary Trials for Inflammatory Bowel Disease: A Systematic Review and Meta-Research Study

First Author: Laura Gregersen

Supplementary material S4: Main outcome measure and findings

Supplementary table S3.1. Primary outcome domain and between-group comparisons of included trials reported by study design.

**Trials with adult participants grouped by study design and diagnose(s) and sorted by publication date.**		
**Parallel designed trials**		
**Study ID, UC participants**	**Primary outcome domain**	**Between-group comparisons**
Alborzi Avanaki 2024	Disease activity	ns
Kedia 2024	Clinical remission	OR 2.9 (1.2-6.7), *p*=0.2 (arm 2 in favour)
Lluansi 2024	Abdominal pain (presence/absence)	ns
Haskey 2023	Dietary adherence	Arm 1 in favour
Miyaguchi 2023	Clinical remission	Arm 1 in favour
Morshedzadeh 2023	Adiponectin concentration	Arm 1 in favour
Kedia 2022	Clinical remission	OR 3.2 (1.1-8.7) (arm 1 in favour)
Keshteli 2022	Clinical relapse	ns
Sarbagili Shabat 2022	Clinical remission	ns
Nyman 2020	Relapse	ns
Bamba 2018	Response to treatment	Not possible to assess
Jian 2018	Disease activity	Arm 1 in favour
Hvas 2016	Clinical remission	ns
Kyaw 2014	Disease activity	Arm 1 in favour
Faghfoori 2011	Inflammatory biomarkers	Not possible to assess
Casellas 2007	Disease activity	Not possible to assess
Seidner 2005	Disease activity	ns
Kato 2004	Response to treatment	Arm 1 in favour
Ishikawa 2003	Exacerbation of patient reported clinical symptoms	Arm 1 in favour
Kanauchi 2002	Disease activity	Arm 1 in favour
Fernandez-Banares 1999	Clinical remission	ns
Almallah 1998	Disease activity	Arm 1 in favour
Candy 1995	Clinical remission	ns
Hawthorne 1992	Clinical remission	ns
Wright 1965	Relapse	Arm 1 in favour
**Study ID, CD participants**	**Primary outcome domain**	**Between-group comparisons**
Lewis 2021	Symptomatic remission	ns: SCD 46.5%; MD 44.5%, p=0.77
Albenberg 2019	Relapse	ns
Gunasekeera 2016	Health related quality of life	Arm 1 in favour

Supplementary material to:

Study Design Complexity and Participant Completion in Dietary Trials for Inflammatory Bowel Disease: A Systematic Review and Meta-Research Study

First Author: Laura Gregersen

Machado 2015	Disease activity	ns
Brotherton 2014	Health related quality of life	Arm 1 in favour
Benjamin 2012	Intestinal permeability	ns: SMD 0.0 (-36.7; 36.7)
Benjamin 2011	Response to treatment	ns
Bartel 2008	Lesions (imaging)	Arm 1 in favour
Eivindson 2005	The insulin growth like factor system	ns
Lomer 2005	Clinical remission	ns
Nielsen 2005	Disease activity	ns
Lomer 2001	Clinical remission	Arm 1 in favour
Den Hond 1999	Intestinal permeability	ns
Ritchie 1987	Withdrawal due to adverse events	Arm 2 in favour
Levenstein 1985	Surgery	ns
**Study ID, mixed IBD participants**	**Primary outcome domain**	**Between-group comparisons**
Liso 2022	Response to treatment	Not possible to assess
Lacerda 2021	Intestinal permeability	Not possible to assess
Cox 2020	GI symptoms improved	Arm 1 in favour
Bodini 2019	Clinical remission	Not possible to assess
Yilmaz 2019	Microbiome	Kefir-groups in favour
Pedersen 2017	IBS-like symptoms improved	OR 5.3 (1.81-15.55), *p*<0.01 (arm 1 in favour)
Brunborg 2008	Joint pain	ns
Bjorck 2000	Extend of acute inflammation (rectal biopsies)	Arm 1 in favour
**Cross-over designed trials**		
**Study ID, UC participants**	**Primary outcome**	**Between-group comparisons**
Laatikainen 2023	Disease activity	ns
Melgaard 2022	Gastrointestinal symptoms	ns
Fritsch 2021	Health related quality of life	Low fat/high fibre diet in favour
Morvaridi 2020	Inflammatory biomarkers	ns
**Study ID, CD participants**	**Primary outcome**	**Between-group comparisons**
Halmos 2016	Microbiome	ns
James 2015	Microbiome	ns
Walters 2014	Microbiome	Arm 2 in favour
Bentz 2010	Health related quality of life	ns
**Study ID, mixed IBD participants**	**Primary outcome domain**	**Between-group comparisons**
Cox 2017	Gastrointestinal symptom relief	Worsening with fructan compared to placebo.

Supplementary material to:

Study Design Complexity and Participant Completion in Dietary Trials for Inflammatory Bowel Disease: A Systematic Review and Meta-Research Study

First Author: Laura Gregersen

**Trials with child participants grouped by study design and sorted by publication date.**		
**Study ID, parallel designed trials**	**Primary outcome**	**Between-group comparisons**
Allen 2022	Acceptability	Not possible to assess
El Amrousy 2022	Clinical remission	ns
Suskind 2020	Clinical remission	Not possible to assess
Strisciuglio 2013	Clinical remission	ns: OR 0.8 (0.2-3.7)
**Study ID, cross-over designed trials**	**Primary outcome**	**Between-group comparisons**
Kaplan 2022	Clinical remission	Not possible to assess
Ejderhamn 1992	Microbiome	Not possible to assess

Study arms correspond to the respective study arm as reported in the main paper Table 1. MD; Mediterranean diet, ns; not significant, OR; odds ratio, SCD; specific carbohydrate diet, SMD; standardised mean difference.

Supplementary material to:

Study Design Complexity and Participant Completion in Dietary Trials for Inflammatory Bowel Disease: A Systematic Review and Meta-Research Study

First Author: Laura Gregersen

Supplementary material S5: Differential attrition sensitivity analyses

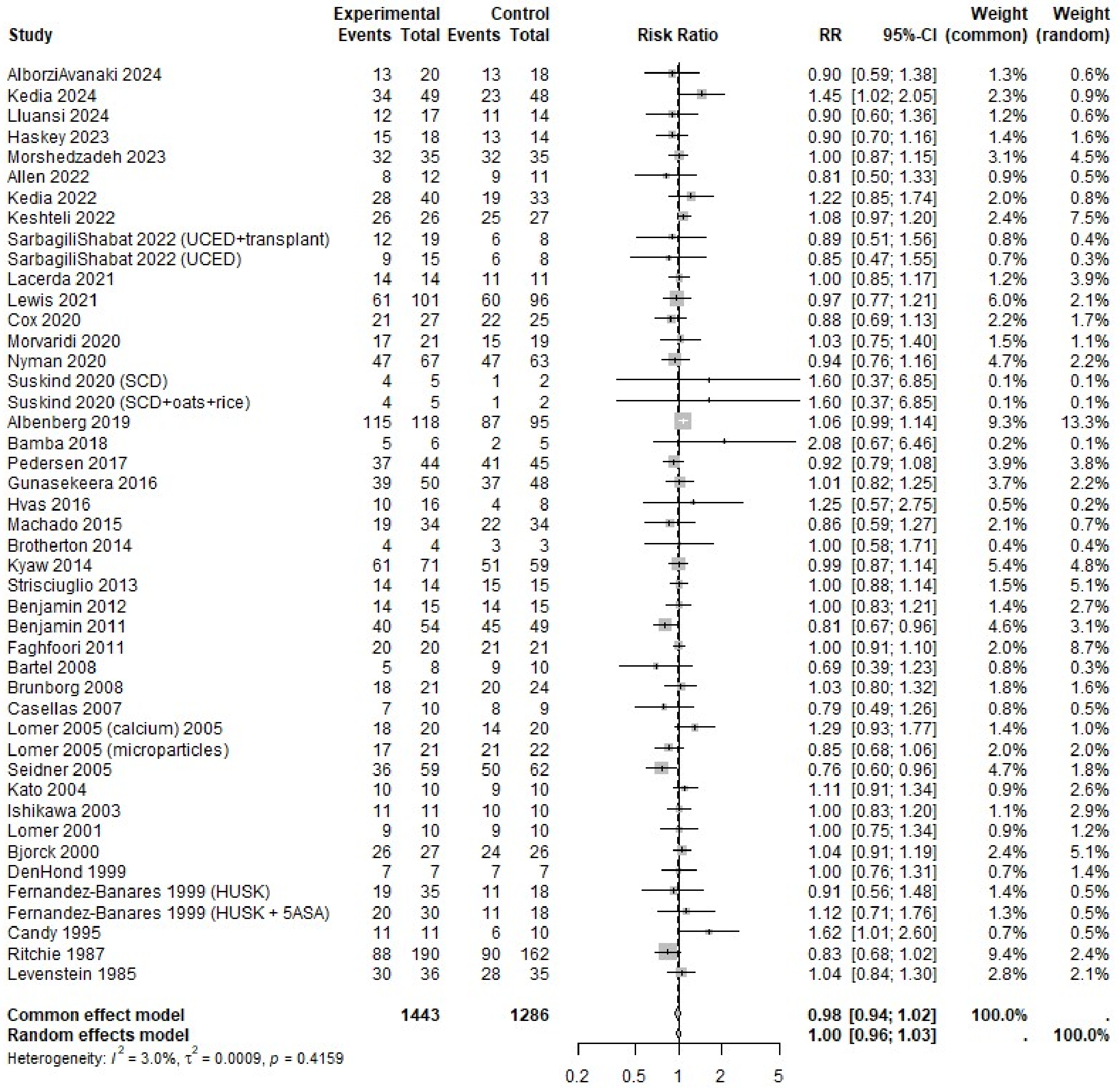

Study	Experimental Events	Experimental Total	Control Events	Control Total	RR	95%-CI	Weight (common)	Weight (random)
AlborziAvanaki 2024	13	20	13	18	0.90	[0.59; 1.38]	1.3%	0.6%
Kedia 2024	34	49	23	48	1.45	[1.02; 2.05]	2.3%	0.9%
Lluansi 2024	12	17	11	14	0.90	[0.60; 1.36]	1.2%	0.6%
Haskey 2023	15	18	13	14	0.90	[0.70; 1.16]	1.4%	1.6%
Morshedzadeh 2023	32	35	32	35	1.00	[0.87; 1.15]	3.1%	4.5%
Allen 2022	8	12	9	11	0.81	[0.50; 1.33]	0.9%	0.5%
Kedia 2022	28	40	19	33	1.22	[0.85; 1.74]	2.0%	0.8%
Keshteli 2022	26	26	25	27	1.08	[0.97; 1.20]	2.4%	7.5%
SarbagiliShabat 2022 (UCED+transplant)	12	19	6	8	0.89	[0.51; 1.56]	0.8%	0.4%
SarbagiliShabat 2022 (UCED)	9	15	6	8	0.85	[0.47; 1.55]	0.7%	0.3%
Lacerda 2021	14	14	11	11	1.00	[0.85; 1.17]	1.2%	3.9%
Lewis 2021	61	101	60	96	0.97	[0.77; 1.21]	6.0%	2.1%
Cox 2020	21	27	22	25	0.88	[0.69; 1.13]	2.2%	1.7%
Morvaridi 2020	17	21	15	19	1.03	[0.75; 1.40]	1.5%	1.1%
Nyman 2020	47	67	47	63	0.94	[0.76; 1.16]	4.7%	2.2%
Suskind 2020 (SCD)	4	5	1	2	1.60	[0.37; 6.85]	0.1%	0.1%
Suskind 2020 (SCD+oats+rice)	4	5	1	2	1.60	[0.37; 6.85]	0.1%	0.1%
Albenberg 2019	115	118	87	95	1.06	[0.99; 1.14]	9.3%	13.3%
Bamba 2018	5	6	2	5	2.08	[0.67; 6.46]	0.2%	0.1%
Pedersen 2017	37	44	41	45	0.92	[0.79; 1.08]	3.9%	3.8%
Gunasekeera 2016	39	50	37	48	1.01	[0.82; 1.25]	3.7%	2.2%
Hvas 2016	10	16	4	8	1.25	[0.57; 2.75]	0.5%	0.2%
Machado 2015	19	34	22	34	0.86	[0.59; 1.27]	2.1%	0.7%
Brotherton 2014	4	4	3	3	1.00	[0.58; 1.71]	0.4%	0.4%
Kyaw 2014	61	71	51	59	0.99	[0.87; 1.14]	5.4%	4.8%
Strisciuglio 2013	14	14	15	15	1.00	[0.88; 1.14]	1.5%	5.1%
Benjamin 2012	14	15	14	15	1.00	[0.83; 1.21]	1.4%	2.7%
Benjamin 2011	40	54	45	49	0.81	[0.67; 0.96]	4.6%	3.1%
Faghfoori 2011	20	20	21	21	1.00	[0.91; 1.10]	2.0%	8.7%
Bartel 2008	5	8	9	10	0.69	[0.39; 1.23]	0.8%	0.3%
Brunborg 2008	18	21	20	24	1.03	[0.80; 1.32]	1.8%	1.6%
Casellas 2007	7	10	8	9	0.79	[0.49; 1.26]	0.8%	0.5%
Lomer 2005 (calcium) 2005	18	20	14	20	1.29	[0.93; 1.77]	1.4%	1.0%
Lomer 2005 (microparticles)	17	21	21	22	0.85	[0.68; 1.06]	2.0%	2.0%
Seidner 2005	36	59	50	62	0.76	[0.60; 0.96]	4.7%	1.8%
Kato 2004	10	10	9	10	1.11	[0.91; 1.34]	0.9%	2.6%
Ishikawa 2003	11	11	10	10	1.00	[0.83; 1.20]	1.1%	2.9%
Lomer 2001	9	10	9	10	1.00	[0.75; 1.34]	0.9%	1.2%
Bjorck 2000	26	27	24	26	1.04	[0.91; 1.19]	2.4%	5.1%
DenHond 1999	7	7	7	7	1.00	[0.76; 1.31]	0.7%	1.4%
Fernandez-Banares 1999 (HUSK)	19	35	11	18	0.91	[0.56; 1.48]	1.4%	0.5%
Fernandez-Banares 1999 (HUSK + 5ASA)	20	30	11	18	1.12	[0.71; 1.76]	1.3%	0.5%
Candy 1995	11	11	6	10	1.62	[1.01; 2.60]	0.7%	0.5%
Ritchie 1987	88	190	90	162	0.83	[0.68; 1.02]	9.4%	2.4%
Levenstein 1985	30	36	28	35	1.04	[0.84; 1.30]	2.8%	2.1%
**Common effect model**		**1443**		**1286**	**0.98**	**[0.94; 1.02]**	**100.0%**	**.**
**Random effects model**					**1.00**	**[0.96; 1.03]**	**.**	**100.0%**

Heterogeneity: $I^2 = 3.0\%$, $\tau^2 = 0.0009$, $p = 0.4159$

Supplementary figure S7.1. Forest plot of the relative risk of completion between study arms per trial, having a parallel study design, and omitting two trials from the main analysis having a very high random weight. RR>1 represents larger completion rates in the experimental arm; RR<1 represents larger completion rates in the control arm. In three trials with two experimental arms compared to one control arm, the control arm was split between the experimental arms.

Supplementary material to:

Study Design Complexity and Participant Completion in Dietary Trials for Inflammatory Bowel Disease: A Systematic Review and Meta-Research Study

First Author: Laura Gregersen

Study	Experimental Events	Experimental Total	Control Events	Control Total	RR	95%-CI	Weight (common)	Weight (random)
AlborziAvanaki 2024	13	20	13	18	0.90	[0.59; 1.38]	1.5%	0.7%
Kedia 2024	34	49	23	48	1.45	[1.02; 2.05]	2.6%	1.1%
Lluansi 2024	12	17	11	14	0.90	[0.60; 1.36]	1.4%	0.8%
Haskey 2023	15	18	13	14	0.90	[0.70; 1.16]	1.6%	2.2%
Morshedzadeh 2023	32	35	32	35	1.00	[0.87; 1.15]	3.6%	6.7%
Allen 2022	8	12	9	11	0.81	[0.50; 1.33]	1.1%	0.6%
Kedia 2022	28	40	19	33	1.22	[0.85; 1.74]	2.3%	1.1%
SarbagiliShabat 2022 (UCED+transplant)	12	19	6	8	0.89	[0.51; 1.56]	0.9%	0.5%
SarbagiliShabat 2022 (UCED)	9	15	6	8	0.85	[0.47; 1.55]	0.9%	0.4%
Lacerda 2021	14	14	11	11	1.00	[0.85; 1.17]	1.4%	5.6%
Lewis 2021	61	101	60	96	0.97	[0.77; 1.21]	6.9%	2.8%
Cox 2020	21	27	22	25	0.88	[0.69; 1.13]	2.6%	2.3%
Morvaridi 2020	17	21	15	19	1.03	[0.75; 1.40]	1.8%	1.4%
Nyman 2020	47	67	47	63	0.94	[0.76; 1.16]	5.4%	3.1%
Suskind 2020 (SCD)	4	5	1	2	1.60	[0.37; 6.85]	0.2%	0.1%
Suskind 2020 (SCD+oats+rice)	4	5	1	2	1.60	[0.37; 6.85]	0.2%	0.1%
Bamba 2018	5	6	2	5	2.08	[0.67; 6.46]	0.2%	0.1%
Pedersen 2017	37	44	41	45	0.92	[0.79; 1.08]	4.6%	5.6%
Gunasekeera 2016	39	50	37	48	1.01	[0.82; 1.25]	4.2%	3.1%
Hvas 2016	10	16	4	8	1.25	[0.57; 2.75]	0.6%	0.2%
Machado 2015	19	34	22	34	0.86	[0.59; 1.27]	2.5%	0.9%
Brotherton 2014	4	4	3	3	1.00	[0.58; 1.71]	0.4%	0.5%
Kyaw 2014	61	71	51	59	0.99	[0.87; 1.14]	6.3%	7.3%
Strisciuglio 2013	14	14	15	15	1.00	[0.88; 1.14]	1.7%	7.8%
Benjamin 2012	14	15	14	15	1.00	[0.83; 1.21]	1.6%	3.8%
Benjamin 2011	40	54	45	49	0.81	[0.67; 0.96]	5.3%	4.4%
Bartel 2008	5	8	9	10	0.69	[0.39; 1.23]	0.9%	0.4%
Brunborg 2008	18	21	20	24	1.03	[0.80; 1.32]	2.1%	2.2%
Casellas 2007	7	10	8	9	0.79	[0.49; 1.26]	0.9%	0.6%
Lomer 2005 (calcium) 2005	18	20	14	20	1.29	[0.93; 1.77]	1.6%	1.3%
Lomer 2005 (microparticles)	17	21	21	22	0.85	[0.68; 1.06]	2.3%	2.7%
Seidner 2005	36	59	50	62	0.76	[0.60; 0.96]	5.5%	2.5%
Kato 2004	10	10	9	10	1.11	[0.91; 1.34]	1.1%	3.6%
Ishikawa 2003	11	11	10	10	1.00	[0.83; 1.20]	1.2%	4.1%
Lomer 2001	9	10	9	10	1.00	[0.75; 1.34]	1.0%	1.6%
Bjorck 2000	26	27	24	26	1.04	[0.91; 1.19]	2.7%	7.8%
DenHond 1999	7	7	7	7	1.00	[0.76; 1.31]	0.8%	1.9%
Fernandez-Banares 1999 (HUSK)	19	35	11	18	0.91	[0.56; 1.48]	1.6%	0.6%
Fernandez-Banares 1999 (HUSK + 5ASA)	20	30	11	18	1.12	[0.71; 1.76]	1.5%	0.7%
Candy 1995	11	11	6	10	1.62	[1.01; 2.60]	0.8%	0.6%
Ritchie 1987	88	190	90	162	0.83	[0.68; 1.02]	10.9%	3.3%
Levenstein 1985	30	36	28	35	1.04	[0.84; 1.30]	3.2%	2.8%
**Common effect model**		**1279**		**1143**	**0.96**	**[0.92; 1.01]**	**100.0%**	.
**Random effects model**					**0.98**	**[0.94; 1.01]**	.	**100.0%**

Heterogeneity: $I^2 = 0.0\%$, $\tau^2 = 0$, $p = 0.5791$

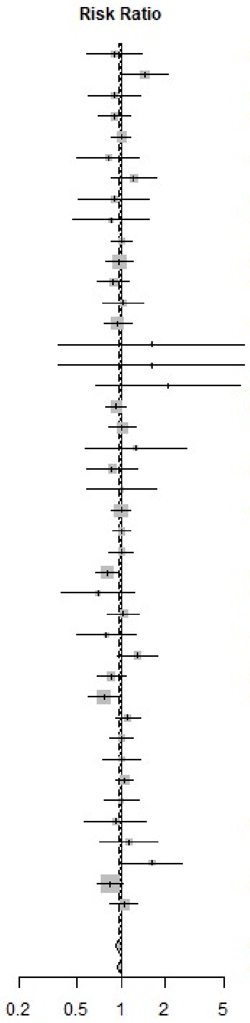

Supplementary figure S7.2. Forest plot of the relative risk of completion between study arms per trial, having a parallel study design, and omitting five trials from the main analysis having a very high random weight. RR>1 represents larger completion rates in the experimental arm; RR<1 represents larger completion rates in the control arm. In three trials with two experimental arms compared to one control arm, the control arm was split between the experimental arms.

Supplementary material to:

Study Design Complexity and Participant Completion in Dietary Trials for Inflammatory Bowel Disease: A Systematic Review and Meta-Research Study

First Author: Laura Gregersen

Supplementary material S6: Risk of bias summary

Risk of bias was evaluated for all included trials. In total, 13 (21%) trials were judged to have a low RoB, 26 (41%) had 'some concerns', and 24 (38%) had high RoB. The lack of a pre-planned analyses was the only reason for an overall judgement of 'some concerns' rather than 'low' in 14 (22%) trials. The domain assessing protocol adherence, i.e., potential non-usage attrition, (domain 2) was judged with 'some concerns' or at 'high risk' in 25 (40%) trials, whereas missing data, often due to dropout attrition (domain 3), raised concerns in 10 (16%) trials. The risk of bias from each domain is summarised in **Supplementary figure S4.1**.

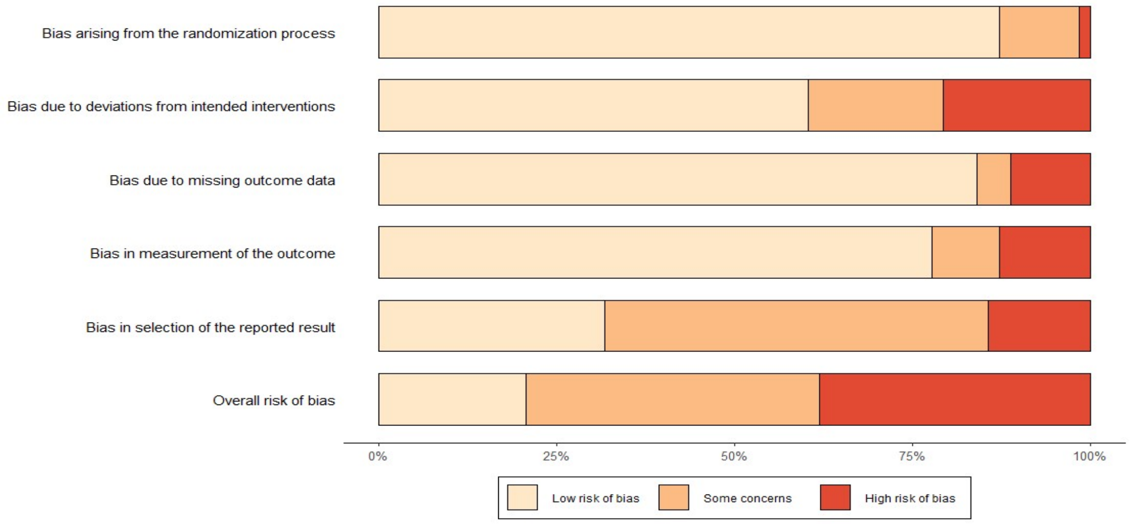

Supplementary figure S4.1. Summary of the risk-of-bias assessment.

Supplementary material to:

Study Design Complexity and Participant Completion in Dietary Trials for Inflammatory Bowel Disease: A Systematic Review and Meta-Research Study

First Author: Laura Gregersen

Supplementary material S7: Stratified sensitivity analyses – results from trials published in 2010-2024

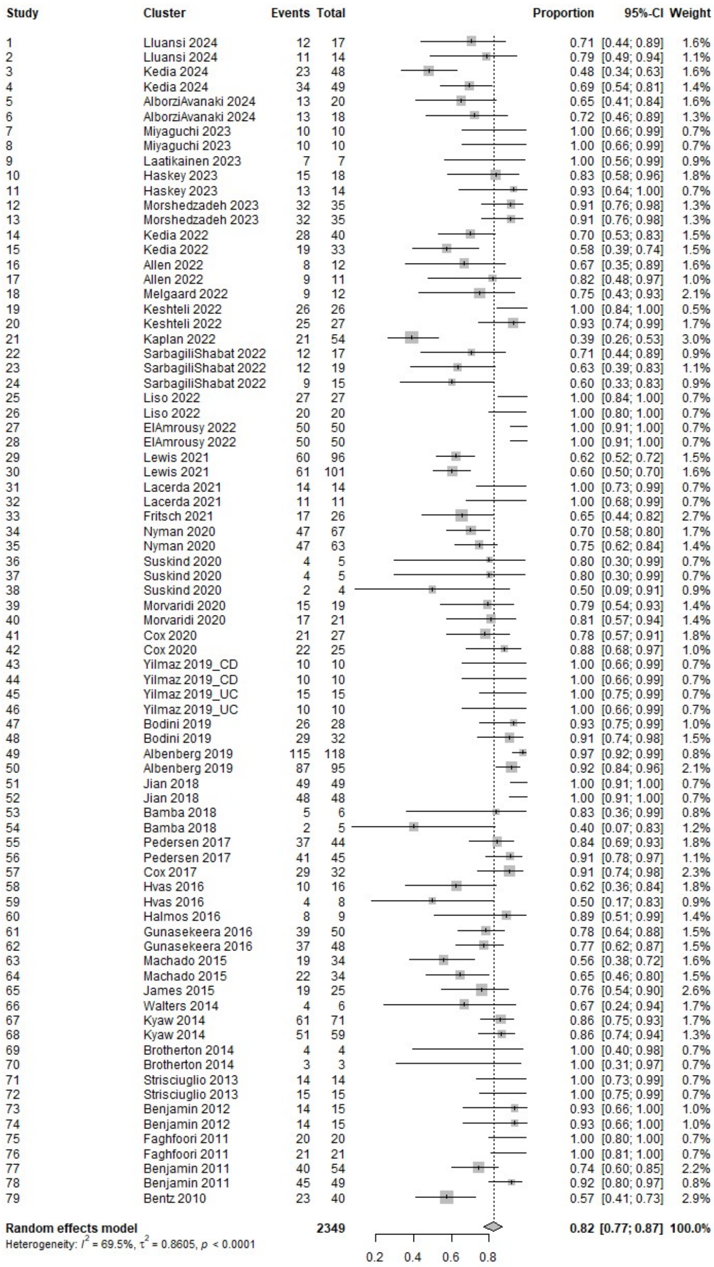

Supplementary figure S5.1. Forest plot of overall completion rate by study arm for the intercept-only model. CD; Crohn's disease, UC; Ulcerative colitis.

Supplementary material to:

Study Design Complexity and Participant Completion in Dietary Trials for Inflammatory Bowel Disease: A Systematic Review and Meta-Research Study

First Author: Laura Gregersen

Supplementary table S5.1. Results of the stratified meta-analysis for design characteristics modifying completion rate (univariate analysis). Fixed effects for trial arms while a random factor for the specific trial.

Analysis	Number of arms (trials)	Completion rate	95% CI		Meta-analysis τ^2	I^2	Risk Ratio (RR) (95%CI)	*P*-value for association
Overall	79 (42)	0.82	0.77	0.87	0.86	70 %		
Diet regimen[†]					0.81	71 %	1.02 (0.94-1.10)	0.33
Restrictive	33 (22)	0.82	0.36	1.00				
Additive	29 (19)	0.80	0.40	1.00				
Study design					0.78	69 %	1.18 (1.11-1.26)	<0.001* (E=1.65)
Parallel	68 (33)	0.85	0.47	1.00				
Cross-over	11 (10)	0.72	0.16	1.00				
Study duration					n.a.	n.a.	n.a.	n.a.
< 4 weeks	1 (1)	1.00	n.a.	n.a.				
≥ 4 weeks	78 (42)	0.82	0.49	1.00				
Faecal samples					0.76	68 %	0.86 (0.76-0.95)	<0.001* (E=1.73)
Yes	57 (32)	0.78	0.52	1.00				
No	22 (11)	0.92	0.19	1.00				
Blood samples					0.81	70 %	1.08 (1.00-1.16)	0.038* (E=1.37)
Yes	62 (33)	0.84	0.45	1.00				
No	17 (10)	0.78	0.16	1.00				
Urine samples					0.76	68 %	1.15 (1.04-1.26)	0.006* (E=1.57)
Yes	5 (3)	0.94	0.03	1.00				
No	74 (40)	0.81	0.48	1.00				
Diet provided					0.77	68 %	0.91 (0.83-0.98)	0.007* (E=1.44)
All/some	45 (25)	0.78	0.45	1.00				
None	34 (18)	0.86	0.27	1.00				
Instructions[‡]					0.80	70 %	1.00 (0.91-1.09)	0.50
By dietician	59 (33)	0.83	0.45	1.00				
None	20 (10)	0.83	0.12	1.00				
Motivation[#]					0.79	67 %	0.93 (0.85-1.01)	0.032* (E=1.36)
Yes	49 (27)	0.80	0.40	1.00				
No/unclear	30 (16)	0.86	0.33	1.00				
Randomisation					0.81	70 %	0.91 (0.82-1.01)	0.031 (E=1.42)
Low RoB	54 (30)	0.80	0.45	1.00				
Some RoB	25 (13)	0.88	0.13	1.00				
Blinding of outcome assessors					0.78	70 %	0.91 (0.84-0.98)	0.006 (E=1.42)
Low RoB	40 (22)	0.79	0.44	1.00				
Some/High RoB	39 (21)	0.86	0.33	1.00				
Handling of missing data					0.76	67 %	0.86 (0.79-0.93)	<0.001* (E=1.60)
Low RoB	19 (11)	0.74	0.25	1.00				
Some/High RoB	60 (32)	0.85	0.46	1.00				
Blinding of participants					0.79	70 %	0.87 (0.80-0.94)	<0.001* (E=1.57)
Yes	34 (20)	0.75	0.48	1.00				
No/unclear	45 (23)	0.87	0.33	1.00				

Variables below the line are domains of risk of bias on the internal validity.[†]; Restrictive diets were defined as those changing habitual diet by restricting specific dietary components or food items, and additive diets were defined as those adding dietary supplements and/or food items to habitual diet, [‡]; diet instructions by other than dieticians/nutritionists excluded, [#]; any non-monetary method used to encourage protocol compliance during participation, *; significant level < 0.05.

Supplementary material to:

Study Design Complexity and Participant Completion in Dietary Trials for Inflammatory Bowel Disease: A Systematic Review and Meta-Research Study

First Author: Laura Gregersen

A

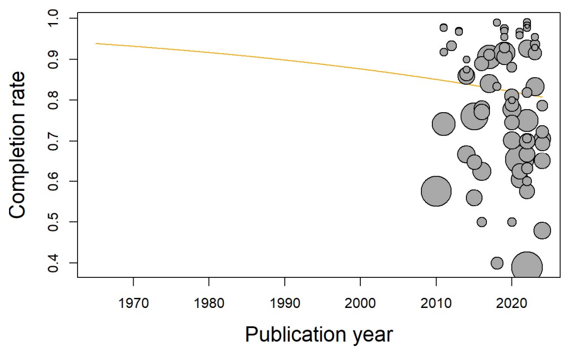

B

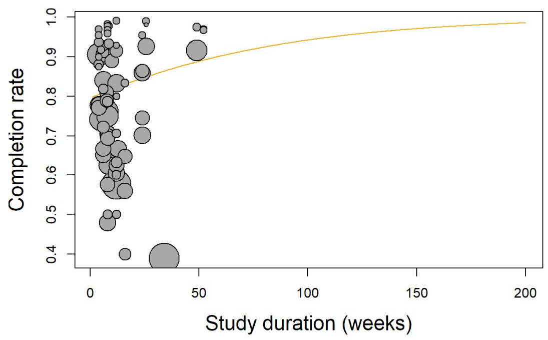

C

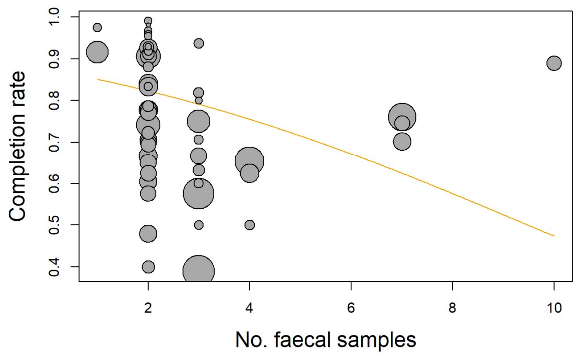

Supplementary figure S5.2. Bubble plot of completion rate according to (**A**) publication year (RR=0.98 (0.90-1.06)), (**B**) study duration measured in weeks (RR=1.01 (0.98-1.05)), and (**C**) the number of faecal samples collected during the trial (RR=0.81 (0.69-0.96), p=0.012, E=1.76), along with the corresponding meta-regression line (orange). The size of the bubbles represents the sample size of each study arm.

Supplementary material to:

Study Design Complexity and Participant Completion in Dietary Trials for Inflammatory Bowel Disease: A Systematic Review and Meta-Research Study

First Author: Laura Gregersen

Supplementary table S5.2. Results of the stratified meta-analysis for explorative study duration measures modifying completion rate (univariate analysis). Fixed effects for trial arms while a random factor for the specific trial.

Analysis	Number of arms	Completion rate	95% CI		τ^2	I^2	Meta-analysis RR (95%CI) ‡	*P*-value for association
Study duration								
≤ 4 weeks	12	0.85	0.35	1.00				
> 4 weeks	67	0.81	0.45	1.00	0.79	96 %	1.05 (0.98-1.12)	0.099
4-8 weeks	26	0.78	0.39	1.00	0.71	68 %	1.10 (0.99-1.21)	0.09
8-12 weeks	21	0.83	0.11	1.00	0.72	66 %	1.03 (0.87-1.19)	0.37
12+ weeks	20	0.84	0.01	1.00	0.79	69%	1.01 (0.83-1.19)	0.46

‡; comparing to studies ≤ 4 weeks, *; significant level < 0.05.

Supplementary material to:

Study Design Complexity and Participant Completion in Dietary Trials for Inflammatory Bowel Disease: A Systematic Review and Meta-Research Study

First Author: Laura Gregersen

Supplementary material S8: Stratified sensitivity analyses – results from trials published before 2010

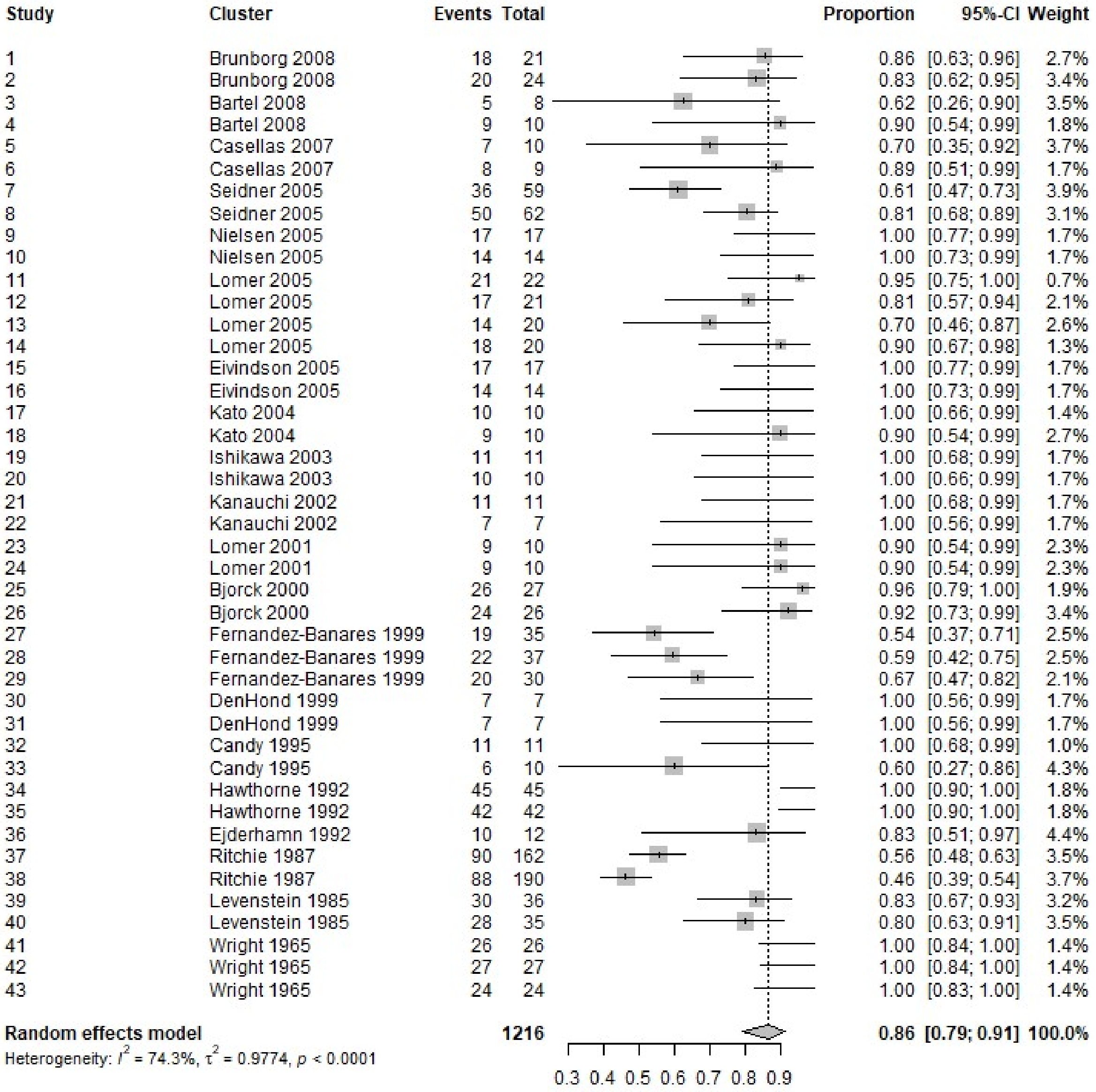

Supplementary figure S6.1. Forest plot of overall completion rate by study arm for the intercept-only model. CD; Crohn's disease, UC; Ulcerative colitis.

Supplementary material to:

Study Design Complexity and Participant Completion in Dietary Trials for Inflammatory Bowel Disease: A Systematic Review and Meta-Research Study

First Author: Laura Gregersen

Supplementary table S6.1. Results of the stratified meta-analysis for design characteristics modifying completion rate (univariate analysis). Fixed effects for trial arms while a random factor for the specific trial.

Analysis	Number of arms (trials)	Completion rate	95% CI		τ^2	I^2	Meta-analysis Risk Ratio (RR) (95%CI)	*P*-value for association
Overall	43 (20)	0.86	0.79	0.91	0.98	74 %		
Diet regimen†					0.86	70 %	0.94 (0.76-1.11)	0.23
Restrictive	14 (7)	0.83	0.00	1.00				
Additive	25 (14)	0.88	0.28	1.00				
Study design					0.92	75 %	1.04 (0.78-1.30)	0.38
Parallel	42 (19)	0.87	0.33	1.00				
Cross-over	1 (1)	0.83	0.00	1.00				
Study duration					0.92	74 %	0.94 (0.80-1.08)	0.20
< 4 weeks	4 (2)	0.82	0.16	1.00				
≥ 4 weeks	39 (18)	0.82	0.29	1.00				
Faecal samples					0.89	74 %	0.86 (0.72-1.01)	0.08
Yes	16 (7)	0.78	0.25	1.00				
No	27 (13)	0.90	0.18	1.00				
Blood samples					0.91	74 %	0.99 (0.78-1.19)	0.45
Yes	36 (16)	0.87	0.29	1.00				
No	7 (4)	0.88	0.00	1.00				
Urine samples					0.84	75 %	0.82 (0.62-1.02)	0.052
Yes	5 (2)	0.72	0.00	1.00				
No	38 (18)	0.87	0.34	1.00				
Diet provided					0.87	67 %	1.04 (0.75-1.32)	0.41
All/some	36 (17)	0.87	0.37	1.00				
None	7 (3)	0.84	0.00	1.00				
Instructions‡					0.90	72 %	0.99 (0.84-1.15)	0.46
By dietician	28 (12)	0.86	0.14	1.00				
None	15 (8)	0.87	0.22	1.00				
Motivation#					0.81	66 %	0.97 (0.84-1.11)	0.36
Yes	28 (12)	0.85	0.12	1.00				
No/unclear	15 (8)	0.87	0.43	1.00				
Randomisation					0.90	68 %	0.94 (0.76-1.13)	0.26
Low RoB	12 (5)	0.83	0.00	1.00				
Some RoB	31 (15)	0.88	0.28	1.00				
Blinding of outcome assessors					0.91	73 %	0.88 (0.72-1.05)	0.07
Low RoB	22 (10)	0.81	0.27	1.00				
Some/High RoB	21 (10)	0.91	0.04	1.00				
Handling of missing data					0.92	74 %	0.88 (0.76-1.01)	0.052
Low RoB	6 (2)	0.74	0.24	1.00				
Some/High RoB	37 (18)	0.88	0.30	1.00				
Blinding of participants					0.76	64 %	1.06 (0.88-1.24)	0.26
Yes	27 (13)	0.88	0.38	1.00				
No/unclear	16 (7)	0.83	0.00	1.00				

Variables below the line are domains of risk of bias on the internal validity.†; Restrictive diets were defined as those changing habitual diet by restricting specific dietary components or food items, and additive diets were defined as those adding dietary supplements and/or food items to habitual diet, ‡; diet instructions by other than dieticians/nutritionists excluded, #; any non-monetary method used to encourage protocol compliance during participation, *; significant level < 0.05.

Supplementary material to:

Study Design Complexity and Participant Completion in Dietary Trials for Inflammatory Bowel Disease: A Systematic Review and Meta-Research Study

First Author: Laura Gregersen

A

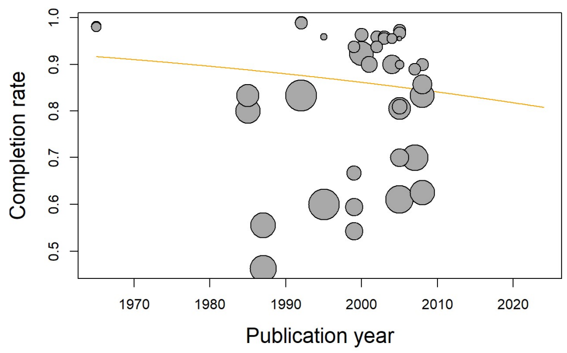

B

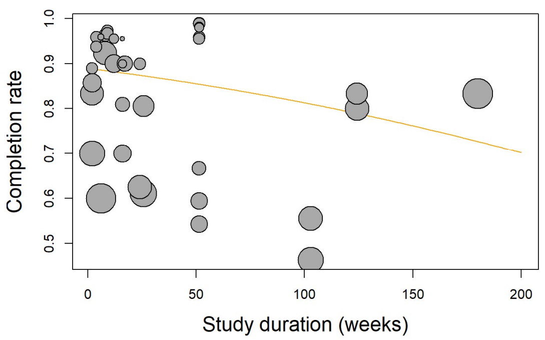

C

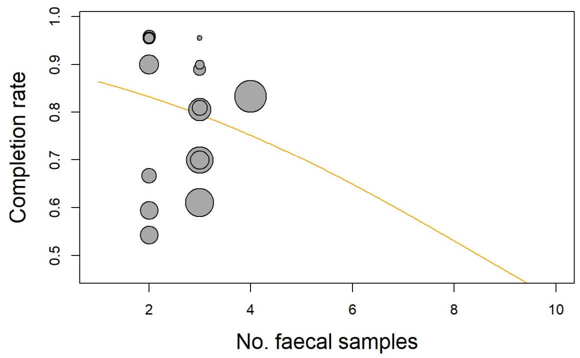

Supplementary figure S6.2. Bubble plot of completion rate according to (**A**) publication year (RR=0.98 (0.93-1.04)), (**B**) study duration measured in weeks (RR=0.99 (0.98-1.00), *p*=0.25), and (**C**) the number of faecal samples collected during the trial (RR=0.78 (0.55-1.12)), along with the corresponding meta-regression line (orange). The size of the bubbles represents the sample size of each study arm.

Supplementary material to:

Study Design Complexity and Participant Completion in Dietary Trials for Inflammatory Bowel Disease: A Systematic Review and Meta-Research Study

First Author: Laura Gregersen

Supplementary table S6.2. Results of the stratified meta-analysis for explorative study duration measures modifying completion rate (univariate analysis). Fixed effects for trial arms while a random factor for the specific trial.

		Meta-analysis						
**Analysis**	**Number of arms**	**Completion rate**	**95% CI**		**τ²**	**I²**	**RR (95%CI)** ‡	***P*-value for association**
Study duration								
≤ 4 weeks	8	0.85	0.26	1.00				
> 4 weeks	35	0.87	0.25	1.00	0.90	73 %	0.98 (0.85-1.12)	0.41
4-8 weeks	4	0.89	0.00	1.00	0.74	71 %	0.96 (0.47-1.46)	0.44
8-12 weeks	6	0.96	0.00	1.00	0.67	69 %	0.89 (0.50-1.28)	0.28
12+ weeks	25	0.84	0.12	1.00	0.72	70%	1.02 (0.85-1.19)	0.41

‡; comparing to studies ≤ 4 weeks, *; significant level < 0.05.
